# Dynamics of ionic liquids under confinement in disordered mesopores

**DOI:** 10.1039/d6na00306k

**Published:** 2026-07-13

**Authors:** Julian Oberdisse, Johan G. Alauzun, Shilpa Sharma, Angel Alegría, Peter Hesemann, Anne-Caroline Genix

**Affiliations:** a Laboratoire Charles Coulomb (L2C), Université de Montpellier, CNRS 34095 Montpellier France anne-caroline.genix@umontpellier.fr; b ICGM, Université de Montpellier, CNRS, ENSCM 34095 Montpellier France; c Department of Polymers and Advanced Materials (EHU), Materials Physics Center (CSIC-EHU) Paseo Manuel de Lardizabal 5 San Sebastián 20018 Spain

## Abstract

The molecular dynamics and phase transitions of ionic liquids confined within mesoporous structures are studied by X-ray diffraction (WAXS), temperature-modulated differential scanning calorimetry (TMDSC), and broadband dielectric spectroscopy (BDS). BMIM-TFSI is introduced into a hybrid ionosilica scaffold *via* a one-pot synthesis, resulting in cylindrical mesopores of average radius between 2 and 3 nm. The thermal properties are monitored as a function of the confinement state, controlled by the amount of incorporated ionic liquid, allowing the construction of an effective phase diagram under confinement. WAXS and TMDSC experiments show that, upon heating, the bulk ionic liquid undergoes cold crystallization followed by melting. Under nanoconfinement, the crystallization temperature shifts to higher values, while the melting temperature shifts downward, until crystallization is completely suppressed under strong confinement. Suppression of crystallization leads to significantly higher conductivity compared to the bulk in the temperature range between cold crystallization and melting, whereas outside this range confinement reduces ionic conductivity. BDS measurements further reveal a strong correlation between ionic conductivity and structural dynamics. The latter is significantly slowed down under confinement and accompanied by an increase in *T*_g_. These results conceptually pave the way to control charge transport by confinement.

## Abstract 2

WAXS, TMDSC, and BDS are used to study ionic liquid confinement in mesoporous ionosilica. Phase behavior, ionic conductivity and structural dynamics are shown to be tunable *via* the degree of confinement.

## Graphical abstract

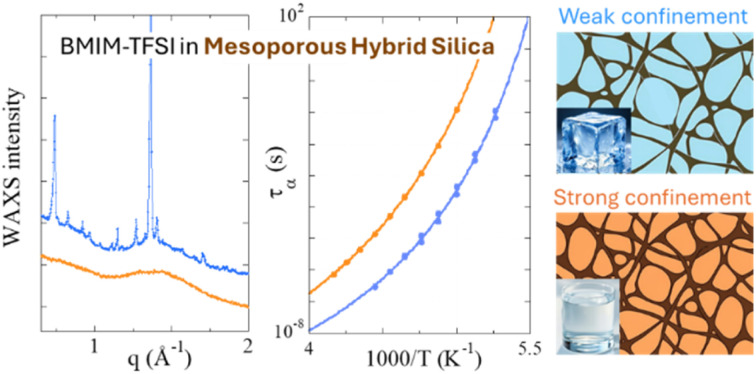

## Introduction

Room-temperature ionic liquids (ILs) have attracted considerable interest over the past few years due to their low melting point (typically below 100 °C), high electrical conductivity, wide electrochemical stability window, and low flammability and vapor pressure.^1,2^ These distinctive physicochemical properties have enabled their use in a broad range of applications, including energy storage,^3,4^ catalysis,^5,6^ and gas adsorption.^7^ At the molecular scale, ILs exhibit a peculiar structural organization governed by strong coulombic interactions, amphiphilicity, and hydrogen bonding.^8,9^ Imidazolium-based ILs, with cations such as 1-alkyl-3-methylimidazolium are a well-studied experimental system,^10^ and various anions have been combined with such cations, inducing ion-specific system properties.^11,12^ For instance, highly fluorinated anions like trifluoromethylsulfonylimide (TFSI) produce water-immiscible, low-hygroscopicity liquids, unlike counter-anions such as nitrate, acetate, or halides.^13^ The phase transitions of imidazolium-based ILs have been investigated by differential scanning calorimetry (DSC).^12,14–16^ In bulk ILs, these studies evidenced the softening of the glassy state at very low temperatures, followed by a cold crystallization with subsequent melting upon heating. The characteristics of these phase transitions depend on the molecular structure, particularly on the number of carbon atoms in the alkyl chains of the cations.^16^ A V-shaped dependence of the melting temperature is observed, reflecting a balance between conformational entropy and enthalpy. This behavior is commonly attributed to the degree of nanostructuration in ILs, arising from the segregation into polar and nonpolar alkyl domains.^16,17^ Such structural features directly impact the transport properties of the ionic liquid, as shown by pulsed-field gradient (PFG) NMR measurements of anion and cation self-diffusion, together with ionic conductivity measurements.^18^

In practical applications involving ILs, maintaining high ionic conductivity under confinement remains challenging, as values up to 10^−2^ S cm^−1^ are typically achieved only in liquid electrolytes, which carry a risk of leakage. Combining the structural stability of mesoporous membranes with the liquid character of confined ILs therefore offers a promising alternative. In this context, it is crucial to investigate how spatial confinement influences both local structure and dynamics to better understand its impact on charge transport. In mesoporous systems, confinement modifies the phase transitions through two competing effects.^19^ First, layering of the ionic liquid near interfaces may promote crystallization and induce an increase in the melting temperature. Secondly, according to Gibbs–Thomson theory, the interfacial energy at the pore surface leads to a depression of the melting point, which becomes more pronounced as the pore size decreases, a trend that has been observed in many studies.^15,20,21^ In some cases, crystallization can be entirely suppressed, as reported for 1-butyl-3-methylimidazolium (BMIM)-TFSI confined within nanoporous alumina with 25 nm pores.^15^ Since crystallization impedes charge transport, ionic conductivity may be significantly enhanced over the temperature range between crystallization and melting, compared to the bulk IL. The specific behavior observed depends on the type of IL, the surface chemistry of the pore walls, as well as particular IL loading protocols, such as under vacuum.^22^ For ILs in amorphous or supercooled state, the effect of nanoconfinement is also complex. The ionic conductivity may either increase or decrease, largely depending on the interactions between the IL and the confining surface.^15,23–25^ IL confined in nanoporous silica membranes has been studied using a combination of PFG NMR and broadband dielectric spectroscopy (BDS).^26^ A significant decrease in the diffusion coefficient compared to the bulk value was observed, while silanization of the pore walls led to only small deviations from the bulk behavior. These changes in translational ion dynamics are accompanied by modifications in the structural relaxation under confinement, as reflected in the glass-transition temperature (*T*_g_).^27^

Ionic liquids can be used in the framework of a one-pot synthesis of mesoporous ionosilica (IS).^28–30^ These are hybrid silica materials in which ionic species are incorporated into the silica network *via* ionic silylated precursors. Acting as a soft template, the IL undergoes arrested phase separation from the growing ionosilica network, generating channels spanning nano- to micrometric length scales. This results in a porous hybrid matrix in which the IL is confined within both meso- and macropores. For BMIM-TFSI, the quantity of meso- and macroporous channels has been determined by us in a recent study combining nitrogen adsorption isotherms with thermogravimetric analysis.^31^ By varying the initial concentration of IL in the synthesis, samples with different degrees of mesoporous confinement can be created. In the present study, the IL concentration is therefore used as main control parameter. Moreover, the geometry of the channels has been measured by small-angle X-ray scattering (SAXS) and described by a model of polydisperse cylinders, where the polydispersity in radius was taken in a quantitative and self-consistent manner from the analysis of the nitrogen adsorption isotherms. As a result, the typical radius was found to decrease from *ca.* 3 to 2 nm as the mesoporous incorporation of ionic liquid increases. This increasing confinement effect will serve as a basis in the present paper. In a follow-up article, we have investigated the structure and conformation of poly(ionic liquid)s embedded in the same system by a combination of small-angle neutron and X-ray scattering.^32^ This additional study has shown that the system is robust, and can accommodate other molecules, including macromolecules, if needed for specific applications.

The goal of the present study is to provide a comprehensive multi-technique characterization of BMIM-TFSI under nanoconfinement, combining structural and transport measurements to elucidate the role of confinement on phase transitions and ionic conductivity. Ionogels of this type are promising candidates for the development of safer, thermally stable quasi-solid electrolytes for electrochemical energy storage devices operating over a wide temperature range. We investigate the phase transitions, ionic conductivity and structural dynamics of BMIM-TFSI confined within hybrid silica pores, comparing these properties with those of the bulk IL under otherwise identical conditions. The hybrid silica used here is an ionosilica, due to the incorporation of ionizable hybrid groups using organosilane precursor molecules. Temperature-dependent wide-angle X-ray scattering (WAXS) is used to identify crystalline or amorphous phases in ILs, with sharp Bragg peaks indicating crystallinity and broad maxima characteristic of amorphous structures. First-order phase transitions are further probed using temperature-modulated DSC and BDS by monitoring the evolution of ionic conductivity with temperature. Combining these techniques provides complementary evidence of the same phase transitions from different approaches. Our results reveal that a crystalline phase can form even deep within the mesoporous system, with crystallization strongly dependent on the degree of confinement and ultimately fully suppressed at the highest confinement. This suppression leads to a conductivity increase of several orders of magnitude within the temperature range between cold crystallization and melting, in agreement with previous observations by Dong *et al.*^15^ in nanoporous alumina. Outside this range, however, confinement reduces ionic conductivity, up to a complete disappearance of the dc conductivity contribution at the highest confinement. In parallel to the translational dynamics associated with conductivity, BDS is moreover used here to probe the reorientational (α) dynamics of the ionic liquid, which is found to slow down under confinement. The α-relaxation times follow the same dynamical trends as ionic conductivity, and the resulting Walden plot evidences a strong coupling between structural relaxation and ionic transport.

## Materials and methods

### Chemicals

The ionosilica precursor tris(3-(trimethoxysilyl)propyl)amine (TTA) was synthesized following previously described protocols.^33^ The imidazolium-based ionic liquid, 1-butyl-3-methylimidazolium bis(trifluoromethylsulfonyl)imide (BMIM-TFSI, >99.5%, *M*_W_ = 419.36 g mol^−1^) was purchased from IoLiTec. The hydrochloric acid was purchased from VWR and used as received. Dry ethanol (≥99.5%) was purchased from Sigma-Aldrich.

### Ionosilica synthesis and templating

The synthesis of binary hybrid ionosilica films (IS/IL) was carried out *via* a one-pot hydrolytic sol–gel process, using the ionic liquid as a soft template, as previously described.^31^ Briefly, the IS precursor (TTA) was mixed with BMIM-TFSI for a few minutes, followed by the addition of ethanol and 1.5 M hydrochloric acid solution. Various TTA-to-IL mixing ratios were used, ranging from 1.91 down to 0.16, corresponding to samples referred to as 1 mL and 12 mL of ionic liquid, respectively, as reported in ref. 31. Gelation resulted from the transesterification and polycondensation of the trimethoxysilyl groups of the TTA, the protonation of which yields the ionic building blocks of the hybrid ionosilica scaffold. The degree of protonation, controlled by the amount of hydrochloric acid, was fixed at 6%. The obtained mixture was cast into a closed Teflon mold with two small holes on the upper side and left at room temperature for 48 hours, forming homogeneous films with tunable thickness. The final materials were dried under reduced pressure (10 mbar) at 100 °C overnight.

The volume fraction *Φ*_IL_ of ionic liquid incorporated in the films was determined by thermogravimetric analysis (TGA, Mettler Toledo, 10 K min^−1^ under air) knowing the density values of each component (*d*_IS_ = 1.54 g cm^−3^, *d*_IL_ = 1.43 g cm^−3^).^31^ The corresponding results are presented in Table 1, with additional samples of similar formulation reported in Table S1. In additional step, the ionic liquid was extracted from the pores using a Soxhlet apparatus with ethanol (IL-solvent) for 48 h. The resulting empty hybrid ionosilica scaffolds were then dried overnight under reduced pressure (10 mbar, 100 °C). The volume fraction of emptied mesopores, *Φ*_meso,_ was estimated from BET analysis.^31^ Increasing the amount of IL in the synthesis results in a larger fraction of confined IL, *Φ*_IL_, and thus a lower amount of ionosilica. In parallel, there is a strong decrease in mesoporosity, while macroporosity increases as *Φ*_IL_ = *Φ*_meso_ + *Φ*_macro_.

**Table 1:** Volume fractions of incorporated ionic liquid and mesopores in binary IS/IL samples with respect to total sample volume (*Φ*_IS_ + *Φ*_meso_ + *Φ*_macro_ = 1). Geometrical pore dimensions, cylinder length and average pore radius, as obtained from the BET/SAXS analysis^31^

Name	*Φ*_IL_ (v%)	*Φ*_meso_ (v%)	*L*_cyl_ (nm)	〈*R*_cyl_〉 (nm)
IS/IL-1	51	38	7	1.8
IS/IL-2	79	30	7	2.1
IS/IL-3	84	14	17	2.9
IS/IL-4	96	7	46	3.0

### Thermal cycle

All samples were dried for 24 h at 100 °C in a vacuum oven prior to measurements and they were subsequently analyzed upon heating from the glassy, amorphous state. Due to technical constraints, cooling rates varied depending on the technique: *e.g.*, 20 K min^−1^ for DSC and 2.6 K min^−1^ for diffraction. This difference did not have any impact, as no crystallization was observed during cooling. During heating, measurements were performed either in a 0.5 K min^−1^ ramp (TMDSC) or at 5 K intervals (diffraction and BDS).

### Structural characterization (WAXS)

X-ray diffraction measurements were performed under high vacuum (10^−5^ mbar) using a high-resolution Malvern Panalytical Empyrean diffractometer operating in reflection mode with Cu Kα_1–2_ radiation (*λ* = 1.5406/1.5444 Å). Flat composite samples were placed on a copper sample holder, 1.8 cm in diameter and 1 mm deep, coated with a nickel layer. The ionic liquid was directly deposited into the holder. Data were collected over a 2*θ* range of 5–40°, with a step size of 0.039° and a total acquisition time of 38 minutes per temperature. This corresponds to a *q*-range of 0.36–2.79 Å^−1^. Each sample was initially measured at 298 K, then rapidly cooled in the cryostat at its maximum achievable rate of 2.6 K min^−1^ down to 123 K. Measurements were subsequently taken at 5 K intervals during the heating process back up to 298 K. Error bars on the transition temperatures determined by WAXS reflect the temperature step of 5 K used in the experiment (±2.5 K).

### Temperature-modulated differential scanning calorimetry (TMDSC)

In TMDSC, a sinusoidal temperature modulation is superimposed on a linear heating rate, allowing the total heat flow to be separated into reversible (heat capacity-related) and non-reversible (kinetic) components. This separation enables the distinction of overlapping thermal phenomena, such as changes in heat capacity and enthalpic relaxation during the glass transition, or melting and recrystallization processes. Temperature-modulated measurements were performed using a Q2000 TMDSC (TA Instruments) with Tzero aluminum pans. Sample masses were between 10 and 15 mg. Prior to measurement, the sample was additionally annealed *in situ* at 373 K for 30 minutes. It was then cooled at a rate of 20 K min^−1^ down to 123 K. The subsequent heating scan was carried out with temperature modulation, starting with a heating rate of 3 K min^−1^ up to 190 K, followed by a slower rate of 0.5 K min^−1^ from 180 K to 288 K, and finally returning to 3 K min^−1^ from 278 K to 373 K. This heating protocol combined different scan rates optimized for the specific thermal transitions, with a slower rate applied in the crystallization and melting regions to ensure accurate characterization, and a faster rate used outside this range, including around the glass transition to improve sensitivity. Overlapping temperature intervals were introduced to ensure continuity between the different heating steps. The modulation amplitude was set to ±0.48 K with a period of 60 seconds. *T*_g_ was determined as the midpoint of the reversible heat capacity change upon heating, with the glass-transition width (Δ*T*_g_) defined as the temperature range between the onset and end points of the transition. Cold-crystallization (*T*_cc_) and melting (*T*_m_) temperatures were obtained from the maximum of their respective transitions in the total heat-flow signal. The uncertainty on all phase transition temperatures determined from TMDSC measurements is ±0.1 K.

### Broadband dielectric spectroscopy (BDS)

BDS measurements were conducted on a broadband high-resolution dielectric spectrometer (Novocontrol Alpha) and a Quatro Cryosystem temperature controller with a stability of ±0.1 K. The complex dielectric permittivity, ε*(*ω*) = *ε*′(*ω*) − *iε*″(*ω*), was measured in the frequency range of 0.1 to 10^7^ Hz (*ω* = 2π*f*) using disk-shaped samples with a diameter of 15 mm and a thickness of the order of 1 mm. The samples were sandwiched between two gold-plated electrodes, forming a capacitor. They were first annealed for at least 1 h at 373 K in the BDS cryostat under nitrogen flow to ensure complete drying, as confirmed by stable conductivity across the probe frequency range. Subsequently, each sample was rapidly cooled in the cryostat to 100 K, and dielectric measurements were performed upon heating in 5 K intervals up to 373 K, followed by a cooling cycle with measurements taken every 5 K down to below *T*_g_. The sample with the lowest ionic liquid content (IS/IL-1) was measured only during the heating cycle.

In BMIM-TFSI, the dielectric response arises from the polarization processes of molecular dipoles and the motion of charge carriers under the alternating electric field, providing access to both dipolar fluctuations and ionic transport dynamics. The BMIM^+^ and TFSI^−^ ions each exhibit moderate intrinsic dipole moments of a few Debye,^34^ while the associated ion pair has an overall dipole moment of the order of 10 D.^35^ The frequency-independent dc conductivity was determined from the plateau of the real part of the conductivity, *σ*′(*ω*), or from the minimum of its logarithmic derivative with respect to ln(*ω*) when the plateau was not well-defined due to contributions from electrode polarization in the low-frequency range.

To determine the relaxation time associated with the structural relaxation dynamics (α-process), we used the dielectric loss modulus, *M*″ = *ε*″/(*ε*′^2^ + *ε*″^2^). This representation is particularly useful in cases of overlapping processes, as it highlights the faster relaxation component that is often hidden in the *ε*″(*ω*) representation. In our case, the α-process is partially masked by electrode polarization on its low-frequency side. Some exemplary dielectric loss functions are shown in Fig. S1. Another point is that the contribution of dc conductivity appears (only) in the imaginary part of ε*(*ω*). This results in an additional term that dominates *ε*″ at low frequencies, taking the form of a 1/*ω* tail. This term can also obscure the α-relaxation peak, especially when ionic conductivity becomes significant (above *T*_g_). To suppress this effect, we used the first-order approximation of the Kramers–Kronig transform,^36^ thereby obtaining a dielectric loss free from dc conductivity contribution (see Fig. S2)1
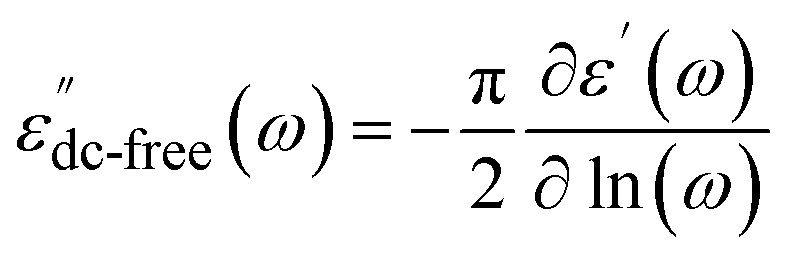


This yields the dielectric loss modulus without the ohmic contribution (see Fig. S3 and S4)2
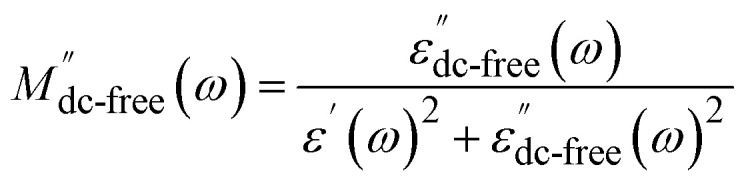


The characteristic relaxation time, *τ*_α_, was determined as the inverse of the angular frequency corresponding to the maximum of the relaxation peak in the 
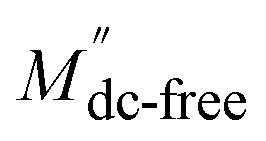
 formalism, *τ*_α_ = 1/(2π*f*_max_). It should be noted that the permittivity-to-modulus transformation may affect the functional shape of the potentially asymmetric structural relaxation, which is typically described by the Havriliak–Negami model in ionic liquids, and slightly shifts the mean relaxation times extracted from *M*″. However, the peak maximum of 
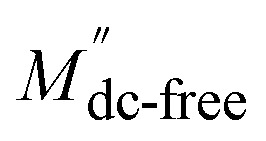
 is used here solely as a consistent and model-free estimate of the characteristic relaxation time, applied identically to all samples for comparative purposes.

## Results and discussion

Before studying the dynamics of the ionic liquid, its phase behavior under nanoconfinement can be investigated by WAXS. In presence of crystallization, multiple and sharp Bragg peaks appear at large angles in X-ray scattering, whereas the amorphous state is characterized by a few broad maxima related to internal interferences of the molecules, as well as weak nearest neighbor correlations. By changing the temperature, these phases with different degrees of order can be evidenced inside the mesopores, while the amorphous hybrid ionosilica scaffold is expected to remain unchanged aside from thermal expansion. Additionally, TMDSC is an effective technique for investigating thermal phase transitions allowing for the detection of subtle changes in the reversible heat capacity associated with the glass transition and melting events. Besides the bulk ionic liquid used as reference sample, four different levels of confinement within mesopores have been studied (Table 1). As described in the Methods section, the lateral confinement in cylindrical mesopores is roughly constant throughout the samples and corresponds to average pore radii between 2 and 3 nm. However, the degree of confinement can be tuned by varying the incorporated amount of ionic liquid, since this directly influences the mesopore fraction. In the samples investigated in this study, the volume fraction of mesopore-confined ionic liquid relative to the total sample volume, *Φ*_meso_, ranges from 7% for the lowest confinement to 38%, as summarized in Table 1.

### WAXS: observation of crystallization and melting

**Fig. 1:**
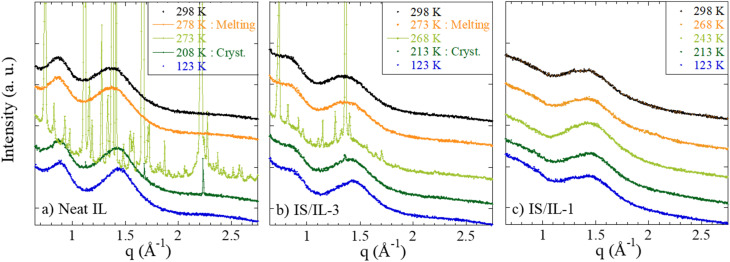
WAXS intensity profiles of BMIM-TFSI under varying confinement conditions: (a) bulk state, (b) intermediate confinement (IS/IL-3, *Φ*_meso_ = 14%), and (c) strong confinement (IS/IL-1, *Φ*_meso_ = 38%), recorded at different temperatures during heating, as indicated in the legend. Intensities are plotted on a linear scale and they are shifted vertically for clarity.

After drying, the samples were cooled below *T*_g_ (see Methods), leading in all cases to the formation of an amorphous, glassy state. The corresponding glass transition will be evidenced later by DSC. The diffraction patterns obtained at selected temperatures during the heating process up to room temperature are shown in Fig. 1. The microscopic behavior of the 1-alkyl-3-methylimidazolium ionic liquid series with TFSI as counter-anion has been deeply investigated at room temperature, as a function of the alkyl chain length on the cation.^10,18,37,38^ The X-ray diffraction patterns of these ionic liquids display three broad peaks in the *q*-range between 0.1 and 2 Å^−1^, as commonly reported for ionic liquids.^9,39^ The lowest-*q* peak shows a strong dependence on the alkyl chain length (see Fig. S5). It is attributed to the formation of nanoscale heterogeneities arising from the segregation of polar and apolar domains within the ionic liquid when the alkyl chains are sufficiently long. In BMIM-TFSI, this feature appears as a shoulder around 0.6 ± 0.1 Å^−1^ (Fig. S5), which is not visible in Fig. 1a, as it lies outside the accessible *q*-range of the diffractometer. At higher *q*, two peaks at 0.87 ± 0.02 and 1.37 ± 0.02 Å^−1^ (Fig. 1a, room temperature) reflect short-range correlations arising from the alternation of oppositely charged ions and correlations between neighboring atoms, respectively. Using the Bragg law, *d* = 2π/*q*, these peaks correspond to characteristic distances of 10.5 ± 1.7, 7.2 ± 0.2, and 4.6 ± 0.1 Å, respectively.

The temperature evolution upon heating is shown for selected samples in Fig. 1. In the bulk sample (Fig. 1a), the onset of crystallization is observed at 208 K by a first Bragg peak added on top of the amorphous structure. At 273 K, the ionic liquid is fully crystallized, as indicated by a series of intense Bragg peaks. This observation is consistent with previous reports of cold crystallization in several ionic liquids, including BMIM-TFSI.^15^ At 278 K, finally, the crystal melts yielding an amorphous liquid of similar structure than the glassy phase, besides thermal expansion slightly shifting the peaks to lower *q*. The temperature dependence of the amorphous peak positions reflects the change in density with temperature, exhibiting a characteristic break in slope at *T*_g_ due to the difference in thermal expansivity below and above the glass transition.^40,41^ For the pure ionic liquid, this analysis yields a WAXS *T*_g_ of 188 ± 5 K (Fig. S6 and details in SI), in good agreement with the calorimetric value reported in the following section. This analysis could not be extended to the ionogels due to the overlap with the ionosilica contribution in the same *q*-range (Fig. S7).

Having established the behavior of the bulk ionic liquid, we now turn to the effect of confinement. Under intermediate confinement as shown in Fig. 1b, a similar sequence of transitions is observed. However, the transition temperatures are shifted towards slightly higher temperatures for the crystallization (213 K), and slightly lower temperatures for the melting (273 K), as can be discriminated given the 5 K temperature step resolution. Another notable difference compared to the bulk sample (Fig. 1a) is that crystallization appears to be incomplete at 268 K. The Bragg peaks are fewer, less intense, and superimposed on a general intensity background given by the two amorphous peaks. This indicates partial crystallization of the ionic liquid, with coexisting amorphous regions. Under the highest confinement (Fig. 1c), the intensity curves at all temperatures exhibit only amorphous features, showing that crystallization is entirely suppressed in strongly confined samples. Finally, the diffraction pattern of the fully amorphous ionosilica (empty scaffold) is given in Fig. S7a, showing a single, weak, and very broad peak centered at *ca.* 1.4 Å^−1^, which remains essentially unchanged with temperature. A comparison of the diffraction patterns at 298 K for the different samples is presented in Fig. S7b. The data show that both amorphous peaks become progressively less well defined with increasing confinement, in particular the peak associated with the periodic arrangement of cations and anions. As the ionic liquid fraction decreases, the contribution from the hybrid ionosilica scaffold becomes increasingly visible, especially for IS/IL-1, which contains about 50% ionosilica.

Here, one may note that the total absence of crystallization under the strongest confinement is a surprising result, given the existence of macropores (13 v% in IS/IL-1). It implies that there are macropores of different sizes. The presence of macropores was deduced from the difference between the total amount of ionic liquid determined by TGA and the volume fraction of mesopores obtained from BET.^31^ In sufficiently small macropores, as observed for IS/IL-1 in Fig. 1c, crystallization appears to be fully suppressed due to confinement. In contrast, in larger macropores approaching considerable fractions of the macroscopic sample, such as for IS/IL-3 in Fig. 1b, portions of the ionic liquid behave similarly to the bulk and crystallize. This subdivision of macropores provides the only rational explanation for the observed behavior. Unfortunately, it does not yield the characteristic length scale separating the two regimes. In the following, we will refer to confinement in mesopores and sufficiently small macropores as a single process, which is dominated by the mesopores, and becomes increasingly important as the mesopore fraction increases.

In summary, confinement of the ionic liquid within the nanometric cylindrical mesopores of hybrid ionosilica reduces crystallization upon heating, eventually leading to its complete suppression. As the fraction of crystallized ionic liquid decreases, the crystallization temperature shifts to higher values, while the melting temperature decreases, giving rise to a “pre-melting” step. While these experiments clearly establish the presence of amorphous and/or crystalline states under varying degrees of confinement, the 5 K temperature steps used here are insufficient to resolve the detailed nature of the transitions, whether they remain abrupt, as in the bulk, or become more gradual. The origin of this confinement effect – whether purely geometrical or due to strong interactions between the ionic liquid and the ionosilica pore walls – also remains unclear. To address these questions, thermal analysis techniques and dielectric spectroscopy will be employed in the following sections.

### TMDSC: a detailed examination of phase transitions

**Fig. 2:**
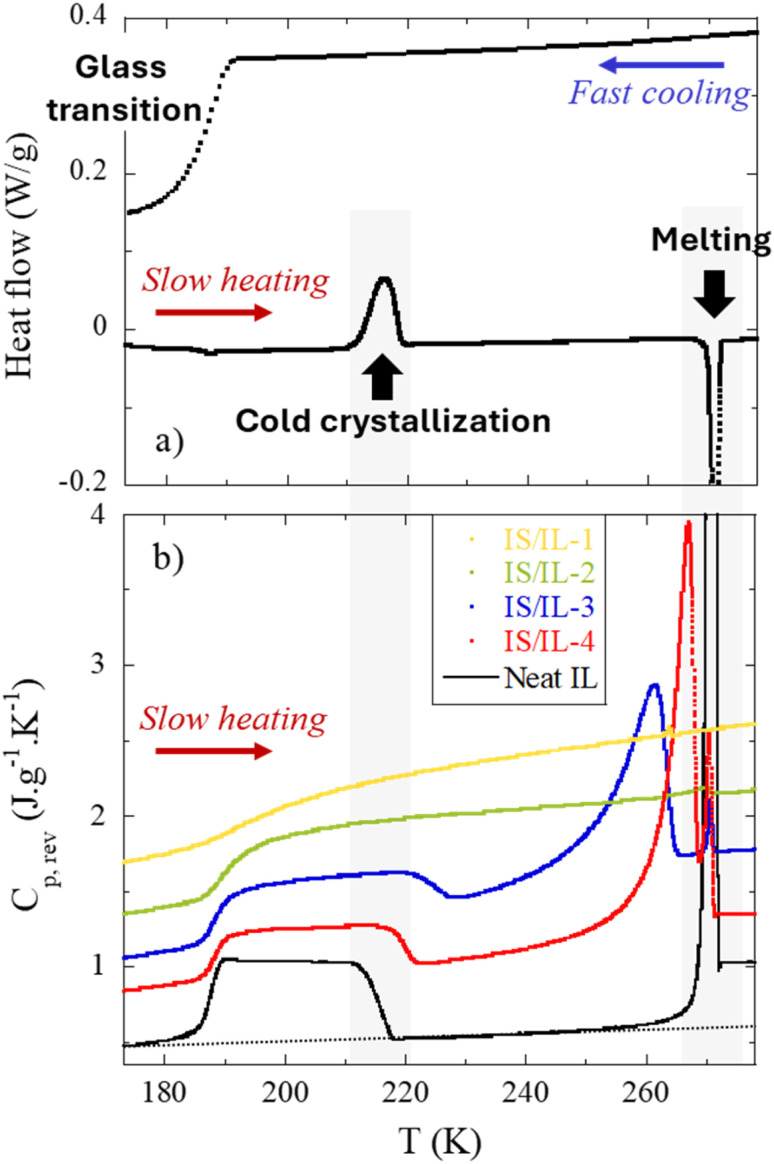
(a) Heat flow as a function of temperature for BMIM-TFSI in the bulk state during cooling (20 K min^−1^) and subsequent heating (0.5 K min^−1^). (b) Reversible heat capacity during heating as a function of temperature for the bulk ionic liquid and under four different confinement conditions (IS/IL curves are shifted for clarity), as indicated in the legend. For BMIM-TFSI, *C*_p_ at 293 K is 1.04 J g^−1^ K^−1^ in agreement with ref. 44.

**Fig. 3:**
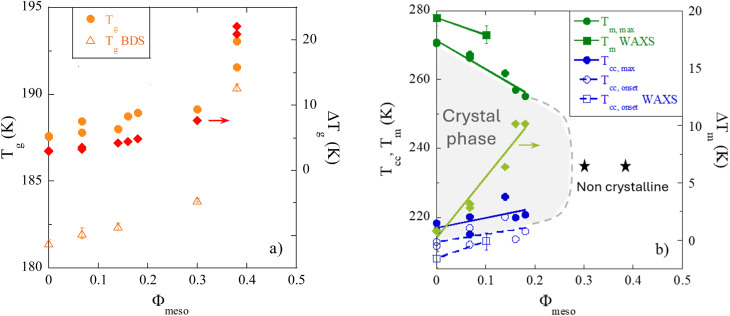
(a) Glass-transition temperature (circles, left axis) and width of the glass transition (red diamonds, right axis) determined from TMDSC as a function of the mesopore volume fraction in IS/IL samples. Additional samples only measured by DSC have been added. The calorimetric *T*_g_ values are compared to values obtained from BDS at *τ* = 100 s (triangles). (b) Effective phase diagram of BMIM-TFSI and IS/IL samples with varying mesopore volume fractions. Temperatures obtained from DSC are shown as circles and those from WAXS as squares. The width of the melting peak is shown on the right axis using diamond symbols. Lines are guides to the eye. The grey region indicates where crystallized BMIM-TFSI is present upon heating, while stars mark conditions where crystallization does not occur.

The heat flow of the bulk ionic liquid is shown in Fig. 2a during fast cooling and subsequent slow heating. The glass transition is clearly visible when it is crossed upon cooling, while no other phase transitions are observed, likely because the sample does not have sufficient time to equilibrate. During slow heating, the glass transition is less pronounced and two thermal transitions are detected: cold crystallization appearing as an exothermic peak, followed by melting at higher temperature, indicated by an endothermic peak. The corresponding temperatures are *T*_cc_ = 216.1 K and *T*_m_ = 270.9 K, in agreement with literature results for BMIM-TFSI,^12,15,20^ with the lower *T*_cc_ being attributed to the extremely slow heating rate employed here (0.5 K min^−1^). In the bottom panel (Fig. 2b), the reversible part of the heat capacity, *C*_p,rev_, under heating is plotted for samples with different degrees of confinement, ranging from bulk to the most strongly confined. The glass transition is evidenced by a sharp increase in *C*_p,rev_, resulting from the enhanced sensitivity of the temperature-modulated signal, and it is observed at 187.6 K in the bulk IL. This is in good agreement with literature data.^12,42,43^ As the amount of ionic liquid decreases (*i.e.*, increasing mesoporosity), the transition becomes progressively broader, indicating the development of a gradient in molecular mobility under confinement. In parallel, *T*_g_ shifts to higher temperatures, reaching 191.5 K in IS/IL-1, which suggests a restriction of molecular motions, typically observed when surface interactions play a major role. This is consistent with the ionic liquid being confined by the surrounding rigid ionosilica walls within the mesopores. Both the evolution of *T*_g_ and Δ*T*_g_ are plotted in Fig. 3a.

Cold crystallization is then visible as a downward step in *C*_p,rev_. Because it involves nucleation and crystal growth, which depend on molecular mobility and time rather than purely on thermodynamic factors, crystallization is a kinetically controlled process. As a result, it appears as an exothermic peak in the non-reversible heat-flow signal. Its indirect signature in the reversible part comes from the lower heat capacity of the crystal (where molecules lose all degrees of freedom) compared to the supercooled liquid, causing a sharp drop in *C*_p,rev_. Extending the low-temperature baseline from below *T*_g_ to above *T*_cc_ (dotted line in Fig. 2b) suggests complete crystallization of the bulk ionic liquid, within the uncertainty of the calorimeter, and assuming *C*_p,glass_ ≈ *C*_p,crystal_. This interpretation is consistent with the diffraction results, which show no amorphous background in the crystallized ionic liquid (Fig. 1a). Under intermediate confinement, the amplitude of the downward step in *C*_p,rev_ is progressively reduced. This step – associated with crystallization – becomes broader, and shifts to higher temperature, as observed for IS/IL-4 and IS/IL-3. The degree of crystallinity of the IL phase, estimated from the enthalpy of crystallization measured in the total heat flow and normalized to the IL weight, are *ca.* 72% and 63% for these samples, respectively. The enthalpy values of the phase transitions are given in Table S2. Then, moving to the two most strongly confined samples (IS/IL-2 and IS/IL-1), the cold crystallization feature disappears completely.

Crystallization is followed by melting. Since the melting process is not clearly separated into reversing and time-dependent contributions, it appears in both the reversible and non-reversible signals. As shown in Fig. 2b, melting mirrors the crystallization behavior under confinement: the melting peak becomes broader and weaker, and it progressively shifts to lower temperatures in IS/IL-4 and IS/IL-3. One can see a strong broadening of the peak toward its low-temperature side. Crystals that were more difficult to develop under confinement (*i.e.*, those with higher *T*_cc_) are therefore less perfect, and they are the first to melt. The crystalline fractions deduced from the total enthalpy of melting give values similar to those obtained from the crystallization peaks (Table S2). Note that a minor fraction of residual ionic liquid, presumably located on the surface of the sample,^20^ undergoes a bulk phase transition at *T* = 270 K in IS/IL-4 and IS/IL-3.

These observations from TMDSC are in line with the diffraction results. The striking result of this series in confinement is that the two phase transitions – cold crystallization followed by melting – are completely suppressed in the samples of highest confinement, IS/IL-2 and IS/IL-1 (*Φ*_meso_ = 30% and 38%, respectively), in close analogy with the structure observed by WAXS in Fig. 1. Cold crystallization remains detectable only under intermediate confinement, where it is hindered and shifted in temperature relative to the bulk. Additional samples of similar formulation were measured to confirm this behavior (Fig. S8a). In Fig. 3b, an effective phase diagram constructed from the results of WAXS (Fig. 1) and TMDSC (Fig. 2) is presented as a function of the control parameter *Φ*_meso_.

The melting temperatures obtained by WAXS and TMDSC are seen to decrease with increasing confinement, whereas the crystallization temperatures increase. Differences between the two techniques arise from the specific point used to identify the transition (onset *versus* maximum), which depends on the resolution of each method. To account for this, Fig. 3b includes the onset of crystallization from DSC (empty circles), which compares well with the temperature at which the first diffraction peak appears in WAXS (empty squares). For melting, there is no well-defined feature in WAXS; therefore, the maximum of the DSC melting peak (full circles) is compared with the temperature at which all diffraction peaks disappear from the WAXS pattern (full squares). Consistently, the latter occurs at a higher temperature. Cold crystallization and melting temperatures are only reported for *Φ*_meso_ below 20 v%, as these phenomena no longer occur at higher mesopore fraction, and in particular at 30% there is no more crystalline phase (stars in Fig. 3b), thereby delimiting the grey zone in Fig. 3b. This grey region indicates the region of low confinement in the effective phase diagram, where the ionic liquid is still able to crystallize within the pores, and subsequently melts. Increasing *Φ*_meso_, and thus the degree of confinement, progressively narrows the temperature range over which the confined IL is crystallized, ultimately leading to the suppression of the phase transitions. Finally, the width of the melting peak is also reported (right axis). In Fig. 2, it was seen to widen with confinement, and indeed there is a strong increase from a narrow peak to a width of about 10 K. The partial suppression of the crystallization under confinement is thus accompanied by a progressive broadening of the melting peak up to its disappearance, indicating a large dispersion in crystal size and quality, arising from the heterogeneity of the mesoporous structure.

It is well established that the phase behavior of a bulk material differs significantly from that of the same material under confinement.^45,46^ At the nanoscale, the influence of crystal size on melting becomes particularly significant, and it is commonly described by the Gibbs–Thomson equation. This relationship predicts a melting-point depression, Δ*T*_m-p_ = *T*_m,bulk_ − *T*_m,confined_, that is inversely proportional to the particle radius, assuming all other parameters to remain constant. As a result, decreasing the crystallite or pore size leads to a corresponding shift of the melting temperature toward lower values. This effect has been observed for several ionic liquids,^47^ including BMIM-TFSI, when confined within controlled-pore glasses^20^ or anodic aluminum oxide (AAO) membranes^15,48^ of various nanopore diameters. In our case, the pore size does not vary substantially across samples (Table 1), particularly among those in which a melting peak is observed (IS/IL-3 and IS/IL-4, see also Table S1). The evolution of *T*_m_ as a function of the inverse pore size is shown in Fig. S8b. While the general trend predicted by the Gibbs–Thomson equation is qualitatively respected, the resulting interfacial tension is anomalously low (see SI), possibly due to the large polydispersity of the small mesopores. A second variable is of importance here: as can be seen in Fig. 3b, the melting-point depression varies proportionally with the mesopore fraction, suggesting that in such disordered mesoporous systems, the mesopore fraction – rather than the average pore size within an intrinsically broad distribution – is the relevant parameter governing melting behavior under confinement. A possible explanation is that most pore radii are below a threshold value inducing strong confinement, and it is the quantity of thereby strongly confined IL which drives the disappearance of the crystal phase. As a result, crystallization is completely suppressed at the highest mesopore fractions, as highlighted by the dashed area in Fig. 3b. This allows the graph to be interpreted as an effective phase diagram as introduced by Dong *et al.*^15,48^

### A step towards applications: improving ionic conductivity by modulating phases *via* confinement

The ionic system studied in this work consists of a hybrid silica scaffold with disordered mesopores formed *via* soft templating by an ionic liquid. The ionic liquid used (BMIM-TFSI) exhibits high ionic conductivity, of the order of 10^−3^–10^−2^ S cm^−1^ at room temperature.^49^ This experimental system is therefore expected to combine high conductivity with a solid-like character due to the encapsulation of the ionic liquid, thereby minimizing the risk of leakage. As described in the Methods section, dielectric spectroscopy is a suitable technique to determine ionic conductivities over a wide temperature range. Moreover, it offers an alternative approach for investigating molecular dynamics *via* the orientational fluctuations of electric dipoles, as opposed to the thermal (enthalpic) signature probed by DSC.

**Fig. 4:**
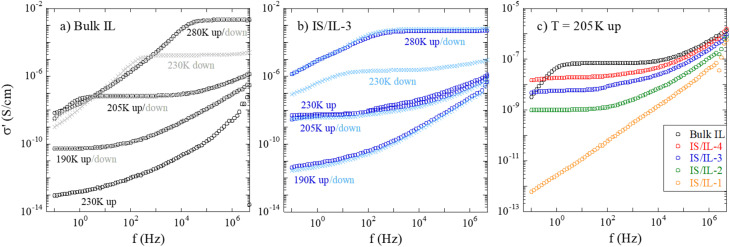
Real part of the complex conductivity as a function of frequency at selected temperatures during heating (circles) and subsequent cooling (crosses) in 5 K steps for (a) bulk ionic liquid and (b) IS/IL-3. (c) Comparison of samples with different confinement degrees at 205 K during heating.

Fig. 4a and b show the real parts of the conductivity functions at different temperatures for BMIM-TFSI in the bulk state and confined within the hybrid ionosilica scaffold, respectively. Note that the conductivity of the empty IS is extremely low (below 10^−14^ S cm^−1^ at room temperature, see Fig. S9a), comparable to that of silica, owing to the small fraction of ionic groups (6%) in the IS used here. This fraction shall be increased in future work. The evolution of *σ*′ in Fig. 4 follows the typical behavior of ion-conducting materials, showing a decrease at low frequencies and high temperatures due to electrode polarization effects.^50,51^ This phenomenon results from the migration and accumulation of ions at the electrode interfaces, leading to the formation of interfacial charge layers that hinder further charge transport. It is primarily associated with the sample-electrode contact rather than an intrinsic property of the material. In the intermediate frequency range, the bulk ionic liquid in Fig. 4a shows a well-defined plateau corresponding to the dc conductivity, *σ*_dc_, which arises from long-range translational motion of the ions. A similar behavior is observed in Fig. 4b for the confined ionic liquid in IS/IL-3. While a weak frequency dependence of the dc plateau (*i.e.*, it is not perfectly flat) has been reported in unidirectional nanoporous silica membranes and attributed to a distribution of pore diameters,^25,26^ this does not appear to be the case here, despite the broad pore size distribution.^31^ Finally, a power-law behavior is observed at high frequencies and low temperatures, reflecting the crossover from diffusive to sub-diffusive dynamics associated with hopping conductivity.

The different samples are compared in Fig. 4c at an intermediate temperature of 205 K where the ionic liquid is in the supercooled state (*T*_g_ < *T* < *T*_cc_). A comparison at a higher temperature of 280 K, corresponding to the liquid state (*T* > *T*_m_), is shown in Fig. S9b. A clear reduction in the plateau level is observed when going from the bulk state to IS/IL-2, corresponding to an increase in both the low-conducting IS fraction and the mesopore volume. This reduction in *σ*_dc_ can be attributed to several factors: the number of ions in the system decreases (less ionic liquid), ions must migrate through increasingly complex and tortuous pathways within the IS scaffold, and some ions may become trapped at the pore interfaces. In the case of IS/IL-1, which exhibits the highest mesoporosity, no dc plateau is observed within the measured frequency range, and the conductivity values are extremely low (below 10^−13^ S cm^−1^). The superposition of *ε*″ and d*ε*′/dln(*f*) for this sample, shown in Fig. S10, confirms that the observed loss arises solely from dielectric relaxation, with no dc-conductivity contribution, as *ε*′ itself does not directly reflect continuous ionic transport. These observations suggest that, under strong confinement, continuous pathways of amorphous ionic liquid may no longer exist for charge transport, and/or that ions are likely blocked within the pores, possibly due to surface effect. The evolution of log(*σ*_dc_) with the amount of ionic liquid entrapped in the composite is shown in Fig. S11a at 205 K and 280 K. It decreases linearly with decreasing the IL fraction, which corresponds to an increasing mesopore fraction (see Table 1 and Fig. S11b for the evolution with *Φ*_meso_), abruptly vanishing at the lowest IL fraction where the mesopore fraction is highest.

The temperature dependence of *σ*_dc_ in Fig. 4a and b shows the expected increase with increasing temperature, except for a significant drop observed in the temperature range of the cold crystallization, as illustrated here at 230 K. This discontinuity in *σ*_dc_ is not observed upon cooling. For the bulk ionic liquid, where crystallization is complete upon heating, ions are blocked within the newly formed crystallites, leading to a pronounced decrease in conductivity and suppression of the dc plateau in the measured frequency range (Fig. 4a). Under confinement in IS/IL-3, which exhibits an IL crystalline fraction of *ca.* 63%, a portion of the ions can still migrate through the amorphous regions, where the presence of crystalline domains interferes with their diffusion. Channels partially blocked by occasional crystallites may further contribute to this behavior. Consequently, the associated conductivity drop in Fig. 4b is reduced by several orders of magnitude compared to the bulk state.

**Fig. 5:**
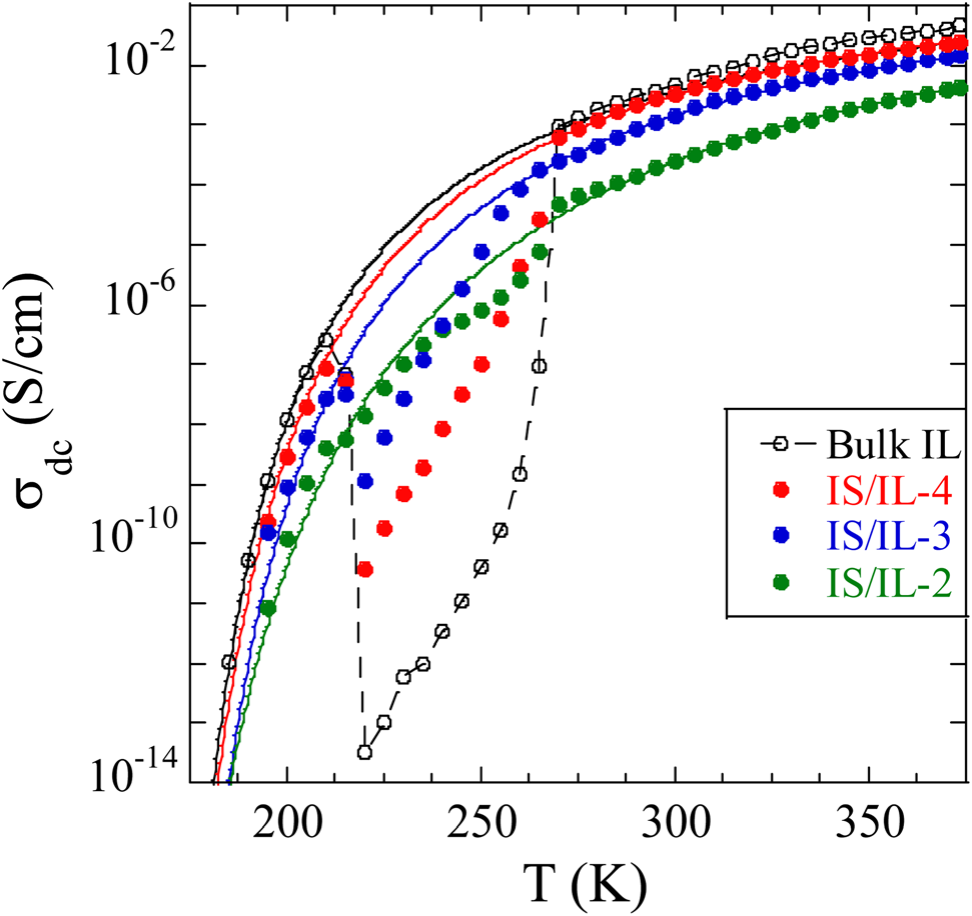
Temperature dependence of the dc conductivity for the bulk ionic liquid and under three different confinement conditions (composites IS/IL), as indicated in the legend. The solid lines correspond to fits of the VFT law in the supercooled state for each sample. The dashed line is a guide to the eye, highlighting the drop in bulk conductivity.

An alternative way to consider these data is to examine the temperature dependence of the dc conductivity for each sample (except the non-conducting IS-IL1). The data obtained during cooling are given in Fig. S12, together with a comparison to literature results for bulk BMIM-TFSI in Fig. S13,^15,52–55^ showing very good agreement. Upon cooling, the ionic liquid remains in the supercooled liquid state without undergoing phase transitions, and the temperature dependence of the dc conductivity is well described by the empirical Vogel–Fulcher–Tammann (VFT) law (see Fig. S12 and Table S3). The data obtained during heating are shown in Fig. 5, allowing a logarithmic-scale comparison of the dc conductivity, which decreases under confinement in the high-temperature range. In this range, the heating data match the cooling data within error bars.

Fig. 5 also highlights the amplitude of the conductivity drop at the crystallization temperature (around 215 K), followed by an increase at the melting temperature (around 270 K), for samples with different levels of confinement. To visualize the magnitude of this drop, one can compare it with the predicted dc conductivity from the VFT law upon cooling, which represents the temperature dependence in the absence of crystallization (solid lines in Fig. 5). Given the strong reduction in conductivity upon crystallization of the bulk ionic liquid, this behavior may represent a significant drawback for any low-temperature application. As the crystallization is partially suppressed under confinement, to an extent that depends on the degree of confinement in IS/IL-3 and IS/IL-4, the conductivity is increased by orders of magnitude between 220 K and 265 K, with respect to the crystallized bulk sample. Although the absolute conductivity values in this temperature range remain too low for energy applications, the pronounced enhancement at low temperatures is encouraging and clearly highlights the beneficial effect of confinement in this range. One may note that a slight reduction in conductivity is still observed for IS/IL-2 around 250 K, although no crystallization was detected from calorimetry for this sample (Fig. 2), most likely due to the higher sensitivity of BDS. This corresponds to a ∼20% decrease in conductivity relative to the corresponding VFT law, although some uncertainty remains in the determination of the dc conductivity for this sample due to the absence of a well-defined plateau in this temperature range upon heating (Fig. S14).

Regarding the temperature value of the IL phase transitions, Fig. 5 shows that the onset of crystallization, located at *T* = 215 K for bulk IL and under intermediate confinement, does not seem to be shifted within the 5 K resolution, whereas the melting transition is reduced from *T*_m_ = 270 K for the bulk IL to 265 K for IS/IL-3. For IS/IL-2 (strong confinement), the crystallization and melting features are poorly resolved, making the transition onsets difficult to identify. In parallel, confinement has a clear effect on the width of the melting transition. For the fully crystallized bulk ionic liquid above 215 K, *σ*_dc_ increases with temperature as expected, followed by a sharp jump at *T*_m_ where it merges with the VFT behavior. In the confined samples, the increase in *σ*_dc_ associated with melting is less abrupt, with the transition occurring over a broader temperature range and gradually merging with the VFT law. A similar trend is observed in the calorimetry results shown in Fig. 2 and 3.

**Fig. 6:**
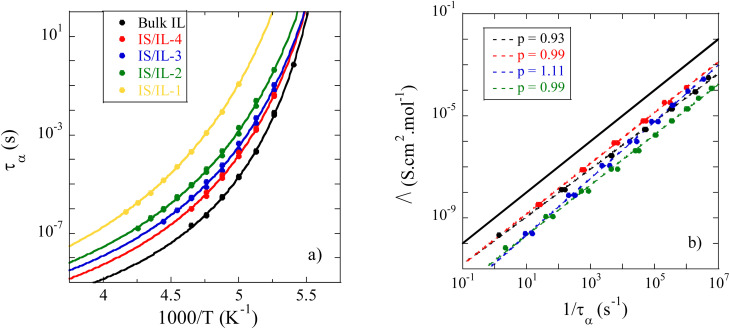
(a) Temperature dependence of the structural relaxation time for the bulk ionic liquid and under four different confinement conditions (composites IS/IL), as indicated in the legend. Solid lines are fits of the VFT law. (b) Modified Walden plot for the same samples. The solid line with slope of 1.0 corresponds to the ideal line of the Walden plot for strong coupling.^57^ Dashed lines are fits by Λ*(*τ*_α_)^*p*^ = constant, with the values of the exponent *p* indicated in the legend.

As a next step in our analysis, we carried out a detailed investigation of the relaxation dynamics of the ionic liquid confined in hybrid ionosilica. To this end, we determined the dielectric loss modulus without the contribution of dc conductivity, as detailed in the Methods section. The data shown in Fig. S3 and S4 reveal a pronounced peak in the temperature range between 185 and 240 K. This peak corresponds to the structural (α-) relaxation, which is associated with the glass transition and involves cooperative molecular motions. As the temperature decreases, this relaxation shifts to lower frequencies. The characteristic relaxation times, determined from the peak position at each temperature, are shown in Fig. 6a for the different samples. As commonly observed for glass-forming liquids,^56^ these data are also well-described by the VFT law, as evidenced by the agreement between the experimental points and the fitted curves. The resulting VFT parameters are provided in Table S4.

A clear slowing down of the structural dynamics is observed under confinement. This is accompanied by an increase in the glass-transition temperature determined by BDS at *τ*_α_ = 100 s (Table S5). A similar behavior was obtained from TMDSC. The *T*_g_ values from both techniques, summarized in Fig. 2a, show the usual shift of a few Kelvin between methods but closely follow the same trend, in particular a strong upturn above *Φ*_meso_ = 30% for the highest confinement. As mentioned above, the increase in *T*_g_ with the mesopore volume fraction suggests attractive interactions between the ionic liquid and the ionosilica pore walls. In parallel, the α-relaxation peak becomes broader, particularly at the highest confinement (IS/IL-1), as shown in Fig. S4. This is consistent with the IL occupying a variety of confined states at high *Φ*_meso_, each contributing on a different time scale to the structural dynamics. Such a behavior highlights the broad distribution of nanoconfinement within these disordered samples, where higher *Φ*_meso_ not only affects a larger proportion of the ionic liquid but also induces a more pronounced slowing down of its molecular dynamics.

From the dc conductivity, the molar conductivity can be calculated as *Λ* = *σ*_dc_/*n*, where *n* is the concentration of free ions contributing to conductivity, given by *n* = *ρ*(*T*)/*M*_w_, with *ρ*(*T*) and *M*_w_ being the temperature-dependent density^53^ and molar mass of the bulk ionic liquid, respectively. The two quantities, *Λ* and *τ*_α_ determined by BDS, can then be used to construct a modified Walden plot, log(*Λ*) *versus* log(1/*τ*_α_), to assess the degree of coupling between ionic transport and structural relaxation.^58^ This representation is shown for the different samples in Fig. 6b, together with the ideal Walden line constructed using literature data for aqueous LiCl, taken as a reference fully dissociated electrolyte in which ion diffusion is controlled by viscosity or structural relaxation time.^57^ In this case, the product Λ*τ_α_ remains constant, resulting in a straight line with unit slope in logarithmic representation. Imidazolium-based ionic liquids typically fall slightly below the ideal line, presumably due to partial ion aggregation.^59^ The same, albeit more pronounced behavior is observed in Fig. 6b for bulk BMIM-TFSI. Note that the data, here, are artificially shifted toward the right (shorter times) because *τ*_α_ was extracted from the dielectric modulus, which shifts the relaxation peak to higher frequency compared to values obtained from the permittivity. Increasing confinement does not appear to modify the exponent *p* in the fitting law Λ*(*τ*_α_)^*p*^ = constant, which remains close to unity within error bars. This indicates that the dynamical coupling between charge transport and structural relaxation is not affected by confinement in hybrid ionosilica. In parallel, the position below the ideal Walden line is not significantly modified under intermediate confinement but seems to shift slightly downward at the strongest confinement (IS/IL-2), possibly reflecting ion immobilization at the pore walls under these conditions.

## Conclusion

The structure and molecular dynamics of BMIM-TFSI confined within hybrid ionosilica mesopores were investigated by WAXS, TMDSC, and BDS. In the bulk ionic liquid, cold crystallization is observed, which impedes ion transport and is therefore detrimental for applications requiring high conductivity below room temperature. Here, we adopt a multi-technique approach to study the effects of confinement, with each method highlighting a different aspect of the phase transitions under confinement.

Throughout this article, the degree of confinement is expressed as the volume fraction of ionic liquid confined within the mesopores, *Φ*_meso_. Previous work combining TGA and BET has shown that part of the ionic liquid is also present in macropores.^31^ As *Φ*_meso_ increases, the volume fraction of macropores decreases significantly, from some 90% to about 13%, while the mesopore radius decreases from about 3 to 2 nm (see Table 1). In all cases, the importance of confinement thus increases with the control parameter *Φ*_meso_. The small remaining fraction of macropores does not seem to play a role. It is possible that surface interactions extend over some (yet unknown) distance into the macropores, thus further reducing their impact.

Our results show that the extent of crystallization strongly depends on the degree of confinement. At intermediate *Φ*_meso_, cold crystallization is partially suppressed and accompanied by a pronounced broadening of the melting transition, suggesting the formation of heterogeneous crystallites. At high confinement, corresponding to *Φ*_meso_ above a critical value of approximately 20–30%, both crystallization and melting are fully suppressed. BDS measurements show that the suppression of crystallization increases ionic conductivity by several orders of magnitude at intermediate temperatures, while confinement reduces it at room temperature and above. Nanoconfinement within the solid and disordered mesoporous matrix thus allows precise control of the phase transitions of the ionic liquid. The techniques employed reveal how molecular mobility is modified by the pore walls, with a significant slowing down of the structural dynamics and an increase in *T*_g_. Importantly, the strong coupling between ionic transport and α-relaxation persists, as shown by the Walden plot, indicating that the underlying mechanism of both processes is unaffected by confinement.

One may note that this study combines methods with molecular resolution, such as WAXS, with macroscopic ones (DSC, BDS). Together, these methods provide a consistent picture of the phase transitions of the ionic liquid under confinement, but each has its strengths and limitations. While the macroscopic techniques provide insight into the phase diagram and molecular mobility, the microscopic one reveals the short-range molecular arrangement naturally adopted by the ionic liquid and how it is modified under confinement.

It is hoped that this fundamental study, with a long-term perspective toward energy applications, will stimulate further research. Possible directions include varying the ionic liquid, particularly its anion, or modifying the hybrid ionosilica to fine-tune surface interactions, and extending the investigation to mechanical properties using Brillouin scattering measurements. Additional techniques, in particular quasi-elastic neutron scattering (QENS), should provide further insight into the dynamics on the nanoscale of specific ingredients of the system, namely the ionic liquid. The system also presents the particularity that the conducting solvent can be extracted by washing with ethanol and subsequently replaced through reimpregnation of the sample. One could make use of the replacement of the conducting liquid either for recycling and waste reduction, or for controlled modifications of the system once the synthesis has been performed with a given ionic liquid. These possibilities are currently under study. Altogether, the IS/IL system can thus be seen as a versatile quasi-solid electrolyte for applications requiring high ionic conductivity without flow or leakage.

## Conflicts of interest

There are no conflicts to declare.

## Supplementary Material

NA-OLF-D6NA00306K-s001

## Data Availability

The data supporting this article have been included as part of the supplementary information (SI). Supplementary information: additional BDS, WAXS, and TMDSC data. See DOI: https://doi.org/10.1039/d6na00306k.
