# Supplementary material for: Dynamics of ionic liquids under confinement in disordered mesopores
Source: Nanoscale Adv. 2026 Jul 13. Online ahead of print. doi: 10.1039/d6na00306k (PMC13382497; doi:10.1039/d6na00306k)
Supplement: NA-OLF-D6NA00306K-s001 [file NA-OLF-D6NA00306K-s001.pdf]

Supplementary Information (SI) for Nanoscale Advances.
This journal is © The Royal Society of Chemistry 2026

Supporting Information

Dynamics of ionic liquids under confinement in disordered mesopores

Julian Oberdisse,[1] Johan G. Alauzun,[2] Shilpa Sharma,[2] Angel Alegría,[3] Peter Hesemann,[2]
Anne-Caroline Genix[1*]

[1]Laboratoire Charles Coulomb (L2C), Université de Montpellier, CNRS, 34095 Montpellier, France

[2]ICGM, Université de Montpellier, CNRS, ENSCM, 34095 Montpellier, France

[3]Department of Polymers and Advanced Materials (EHU), Materials Physics Center (CSIC-EHU), Paseo Manuel de Lardizabal 5, San Sebastián 20018, Spain

* Corresponding author: anne-caroline.genix@umontpellier.fr

1. Additional BDS data

Some exemplary real (ε') and imaginary (ε'') parts of the dielectric permittivity are shown in Figure S1 over different temperature ranges with respect to the phase transitions. In the temperature range between T_g and T_{cc} (Figures S1a and S1b), the α-process of the ionic liquid is partially masked by the strong low-frequency increase arising from electrode polarization, as well as by the conductivity contribution. By calculating the dc-free dielectric loss from the real part of the dielectric response (eq 1), the contribution from dc conductivity can be suppressed, thereby enhancing the visibility of the α-relaxation peak on the high-frequency side of the electrode polarization contribution as shown in Figure S2a (see arrows). With increasing temperatures, there is the range where the ionic liquid phase is partially crystalline (240 K, Figures S1d and S2b). The α-relaxation shifts toward higher frequencies and it is outside the measurement window at 280 K (Figures S1f and S2c), where only the electrode polarization process remains visible within the frequency window.

It should be noted that the determination of the Kramers-Kronig-derived $\varepsilon''_{dc\text{-}free}$ is strictly valid for ohmic conductivity only; non-ohmic conductivity or electrode polarization contributions may introduce anomalies in the spectral shape. Additionally, the derivative of ε' may become unreliable at the low- and high-frequency boundaries of the experimental window, leading to a slight degradation of the quality of the derived $\varepsilon''_{dc\text{-}free}$ data. However, these shortcomings do not affect the quality of our analysis, which is exclusively centered on the determination of a representative peak frequency.

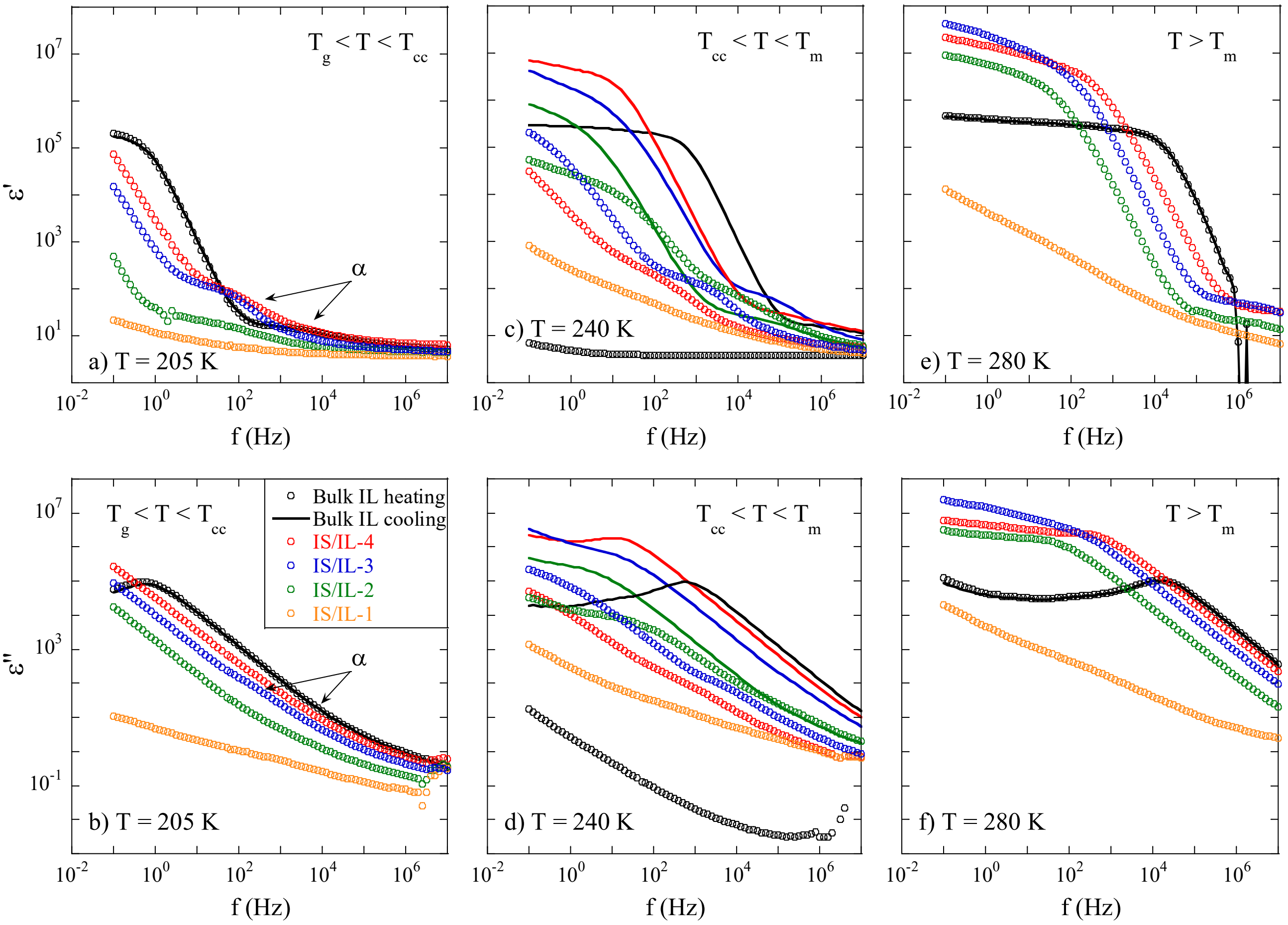

Figure S1. Frequency dependence of the real and imaginary parts of the complex permittivity at 205 K **(a: ε' – b: ε'')**, 240 K **(c: ε' – d: ε'')** and 280 K **(e: ε' – f: ε'')** for bulk BMIM-TFSI and under different confinement conditions (IS/IL composites), as indicated in the legend. Empty symbols correspond to measurements upon heating, while solid lines represent measurements upon cooling, where no crystallization is observed. Arrows in **a)** and **b)** indicate the position of the structural (α) relaxation in the pure ionic liquid and in the ionogels.

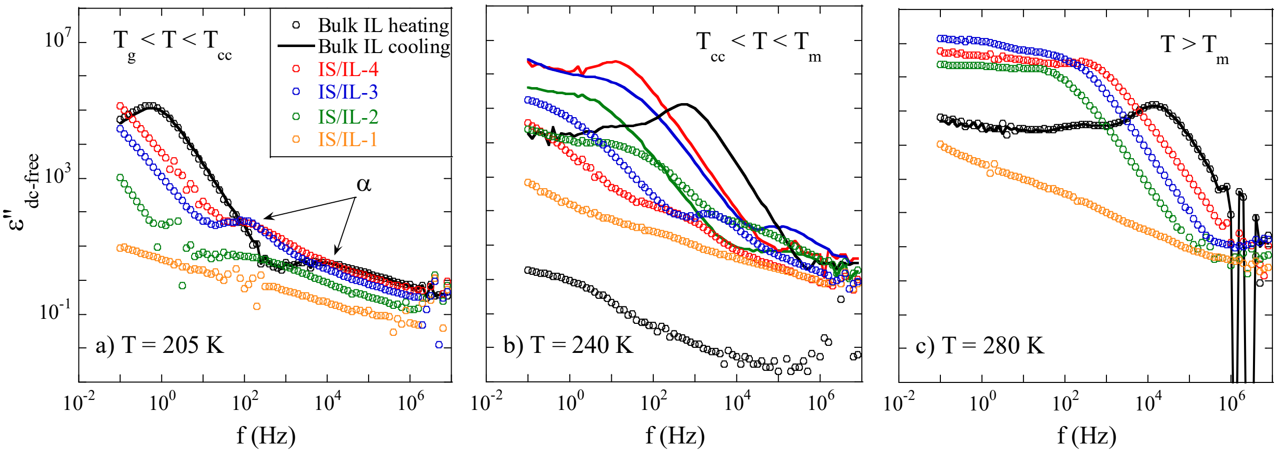

Figure S2. Frequency dependence of the imaginary part of the complex permittivity without the contribution of dc conductivity, $\varepsilon''_{dc\text{-}free}$, at 205 K **(a)**, 240 K **(b)** and 280 K **(c)** for bulk BMIM-TFSI and under different confinement conditions (IS/IL composites), as indicated in the legend. Empty symbols correspond to measurements upon heating, while solid lines represent measurements upon cooling, where no crystallization is observed. Arrows in **a)** indicate the position of the structural (α) relaxation in the pure ionic liquid and in the ionogels.

Finally, it is also convenient to analyze the data in terms of the loss modulus M'', which emphasizes the contribution of faster dynamical processes. In this representation, the relative proportions of the different contributions are modified: slower processes, such as electrode polarization, become much less dominant, while the α-relaxation is comparatively enhanced. The M'' spectra, shown with and without the dc contribution (eq. 2), are shown in Figure S3 for the bulk IL at different temperatures. At low Ts in Figure S3a, within the range of the secondary relaxations as previously observed in imidazolium-based ionic liquids [1-3] and typical of glass-forming systems, the spectra overlap indicating the absence of a measurable dc contribution. This contrasts with the α-relaxation region in Figure S3b, where a dc contribution is clearly present. Note that the tail of the highest-frequency secondary relaxation is still visible at 185 K and 190 K on the high-frequency side of the α-process.

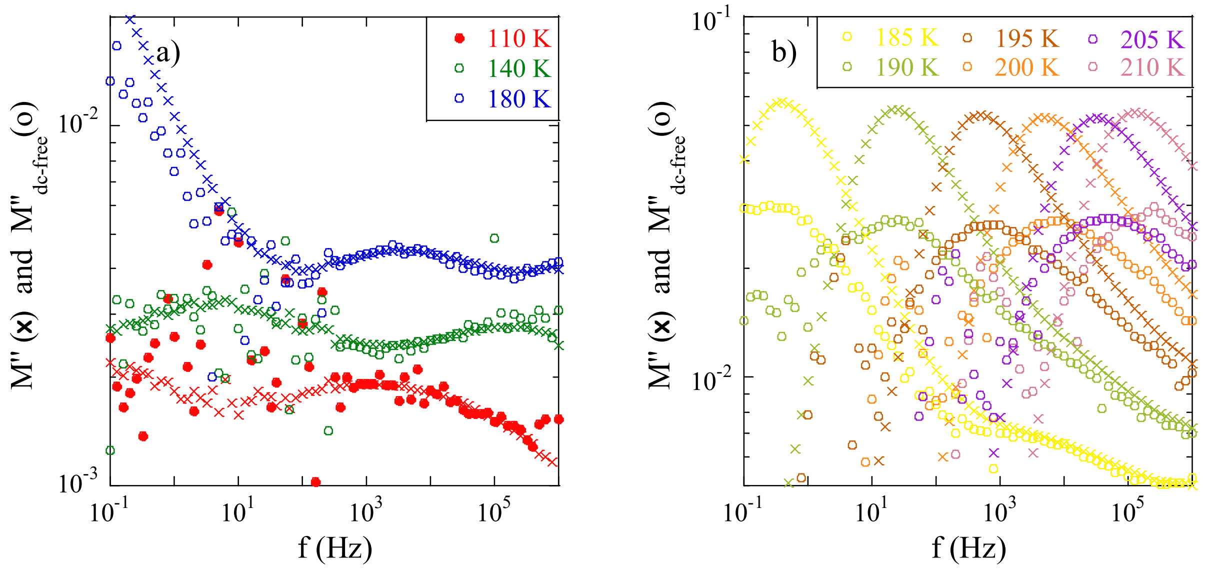

Figure S3. Frequency dependence of the imaginary part of the complex modulus with (M'', crosses) and without ($M''_{dc\text{-}free}$, circles) the dc contribution for bulk BMIM-TFSI during heating, in the temperature range of the secondary relaxations **(a)** and the α-relaxation **(b)**.

Figure S4 compares both types of moduli for the different samples. In Figure S4b, the spectra for IS/IL-1 superimpose within error bars, demonstrating that the dc contribution is negligible for this sample.

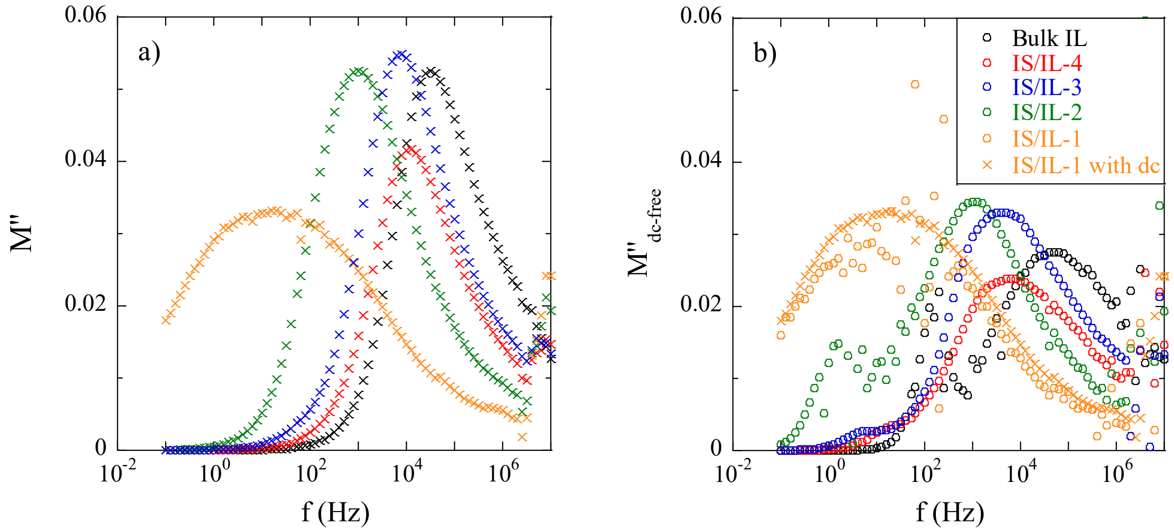

Figure S4. Frequency dependence of the imaginary part of the complex modulus, M'' **(a)** and of the dc-free modulus, $M''_{dc\text{-}free}$ **(b)** at 205 K during heating for the bulk IL and under different confinement conditions (IS/IL composites), as indicated in the legend. Crosses in (b) correspond to the M'' spectrum shown in (a) for IS/IL-1.

2. Additional WAXS data

WAXS data of the 1-alkyl-3-methylimidazolium-TFSI ionic liquid series (Figure S5), measured on the SWING beamline at synchrotron SOLEIL, show three broad peaks between 0.1 and 2 Å^{-1}. The lowest-q peak shifts with alkyl-chain length, reflecting nanoscale segregation of polar and apolar domains for longer chains. This peak falls outside the measurable range of the diffraction experiments shown in Figure 1 at different temperatures.

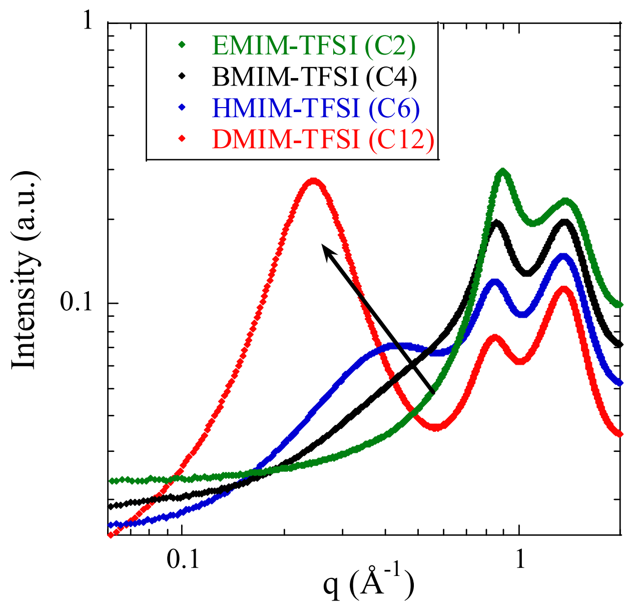

Figure S5. Room-temperature WAXS data of bulk 1-alkyl-3-methylimidazolium-TFSI ionic liquids with different alkyl side-chain lengths, C_n, as indicated in the legend. BMIM-TFSI used in this study is shown in black. Intensities are vertically shifted for clarity. The arrow highlights the shift of the lowest-q peak with increasing alkyl-chain length.

Figure S6a shows the WAXS intensity profile of pure BMIM-TFSI at 133 K, fitted using the sum of two Gaussian functions and a power-law background over the q-range 0.65 – 2.5 Å^{-1}. From these fits, performed at each temperature in the range where the IL is in amorphous state, the positions of the two diffraction peaks associated with short-range ionic correlations were extracted. For each peak, the q-position was normalized to its value at the lowest measured temperature: $q_{norm}(T) = q_{max}(T)/q_{max}(123K)$. The resulting temperature dependence of both normalized peak positions is shown in Figure S6b. From the break in slope, which appears at the same temperature within error bars, one can deduce a T_g of 188 ± 5 K for BMIM-TFSI.

This analysis was performed only for the pure ionic liquid. In the case of the ionogels, the molecular correlations of the ionosilica matrix overlap with those of the ionic liquid in this q-range (Figure S7). Combined with a different temperature dependence of the ionosilica contribution, which remains well below its own T_g over the investigated temperature range, this overlap does not allow a reliable determination of T_g from the WAXS data of the ionogels.

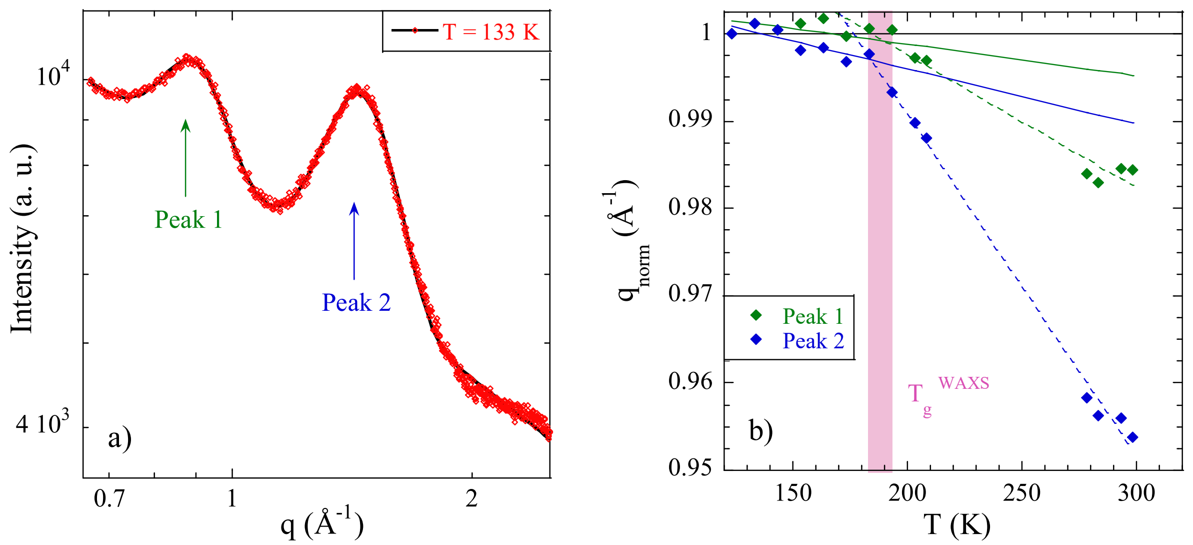

Figure S6. **a)** WAXS intensity profile of pure BMIM-TFSI at 133 K. The black solid line is a fit using the sum of two gaussian peaks and a power law background. **b)** Temperature dependence of the normalized peak position for the two diffraction peaks shown in **a)**. Solid and dotted lines are linear fits highlighting the break in slope.

The diffraction pattern of the empty ionosilica scaffold, after removal of the ionic liquid by washing with ethanol, is shown in Figure S7a. The absence of sharp diffraction peaks indicates that the material is fully amorphous. A comparison of the different samples measured by WAXS is shown in Figure S7b.

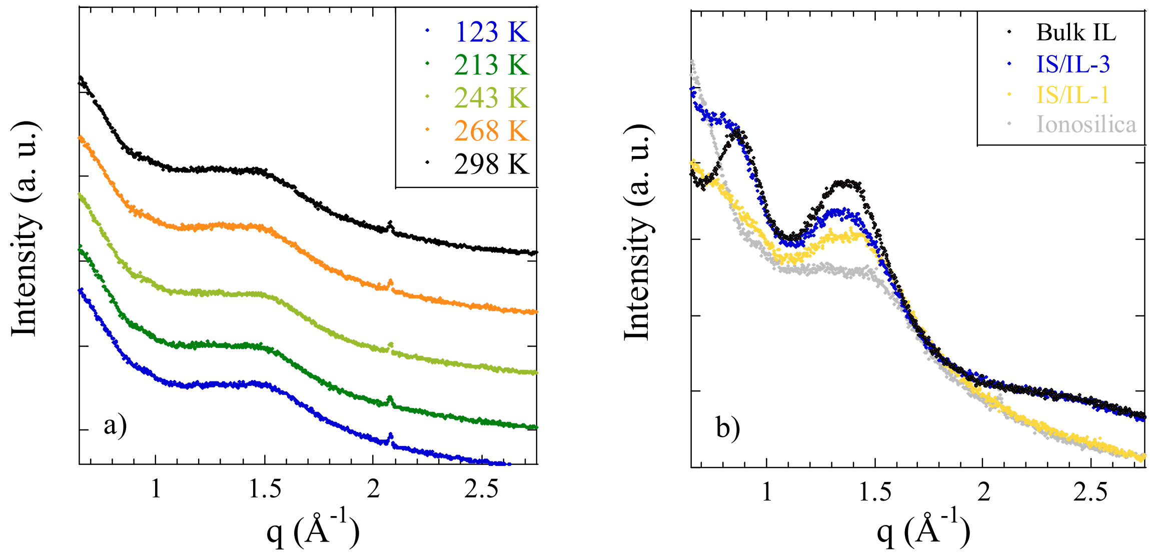

Figure S7. WAXS intensity profiles plotted on a linear scale and shifted vertically for clarity. (a) Empty ionosilica scaffold recorded at different temperatures during heating, as indicated in the legend. (b) Room temperature profiles of bulk BMIM-TFSI, IS/IL composites under two confinement conditions, and the ionosilica scaffold, as indicated in the legend.

3. Additional TMDSC data

The calorimetry data for two additional samples with compositions close to IS/IL-3 (Table S1) are shown in Figure S8a, together with those of IS/IL-3.

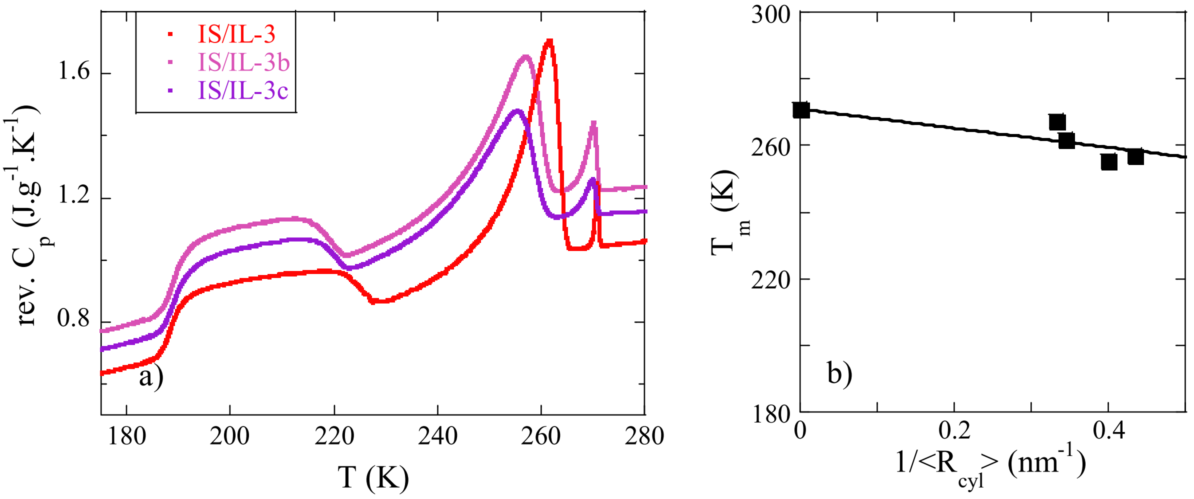

Figure S8. **a)** Reversible heat capacity during heating as a function of temperature for confined ionic liquids (IS/IL curves are shifted for clarity), as indicated in the legend. **b)** Melting temperature determined by DSC as a function of the inverse pore radius for the ionogel samples. The solid line is a fit to eq. S1.

Table S1 reports the geometrical pore dimensions derived from the combined BET/SAXS analysis for these samples [4], including the cylinder length and average pore radius. The average pore radius accounts for polydispersity and the cylinder length is the only fitted parameter, with an estimated uncertainty of a few percent.

Table S1. Volume fractions of incorporated ionic liquid and mesopores in binary IS/IL samples with respect to total sample volume ($\Phi_{IS} + \Phi_{meso} + \Phi_{macro} = 1$). Geometrical pore dimensions, cylinder length and average pore radius, as obtained from the BET/SAXS analysis [4].

Name	Φ_{IL} (v%)	Φ_{meso} (v%)	L_{cyl} (nm)	<R_{cyl}> (nm)
IS/IL-3	84	14	17	2.9
IS/IL-3b	86	16	14.5	2.3
IS/IL-3c	85	18	12	2.5

The melting temperature T_m of IL crystals confined in cylindrical pores of radius R can be expressed using the Gibbs–Thomson equation:

$$T_m(R) = T_{m,bulk}\left(1 + \frac{2\,\sigma_{cl}\cos\theta}{\Delta H_m\,\rho_{IL}}\,\frac{1}{R}\right) \qquad S1$$

where $T_{m,bulk}$ = 270.9 K is the melting temperature of the bulk IL, ΔH_m = 46 J/g the specific enthalpy of melting (Table S2) and ρ_{IL} the density. σ_{cl} is the surface tension between crystal and liquid and θ is the contact angle between the IL crystal and the IS scaffold. Following the usual assumption: θ = 180° (no wetting). [5] The T_m values determined from the DSC scans are plotted as a function of the inverse average pore radius <R_{cyl}> in Figure S8b. The surface tension obtained from the fit of these data to eq. S1 is σ_{cl} = 3.5 mN/m, which is significantly lower than the value reported by Dong et al (σ_{cl} = 30 mN/m) for BMIM-TFSI confined in nanoporous AAO membranes. [5] We attribute this discrepancy to the broad pore size distribution within the mesopore range. [4] Using the average pore radius alone is thus not representative of the overall behavior.

The enthalpy changes for cold crystallization and melting are given in Table S2 for all samples except IS/IL-2 and IS/IL-1, for which cold crystallization is completely suppressed. The specific enthalpy (J/g, per unit mass of sample) obtained directly from the total DSC heat flow upon heating is converted to

molar enthalpy of IL (J/mol) by dividing by the mass fraction of BMIM-TFSI and multiplying by its molar mass (419.36 g/mol). The degree of crystallinity χ_c of the ionic liquid phase is determined as the ratio of the molar enthalpy to that of the bulk BMIM-TFSI, where the bulk ionic liquid is considered to be fully crystalline (as discussed in the article), measured under identical conditions.

Table S2. Weight fractions of incorporated ionic liquid in binary IS/IL samples, specific and molar enthalpies of cold crystallization and melting, and degree of crystallinity of the IL phase, for samples exhibiting cold crystallization.

Name	Φ_{IL} (w%)	ΔH_{cc} (J/g)	ΔH_{cc} (kJ/mol of IL)	χ_c from cryst.	ΔH_m (J/g)	ΔH_m (kJ/mol of IL)	χ_c from melting
BMIM-TFSI	100	35	15	100%	46	19	100%
IS/IL-4	95	24	11	72%	30	13	69%
IS/IL-3	82	18	9	63%	20	10	53%
IS/IL-3b	84	14	7	48%	18	9	47%
IS/IL-3c	84	13	6	44%	14	7	36%

4. Additional BDS data – Conductivity

The conductivity measurements of the empty IS scaffold (i.e., IS/IL after ethanol washing to remove the ionic liquid from the pores) are shown in Figure S9a. As can be seen, the IS conductivity is extremely low, which is attributed to the small fraction of ionic building blocks (only 6% TTA protonation). A comparison of the conductivities of the different samples above the melting temperature of the ionic liquid is presented in Figure S9b.

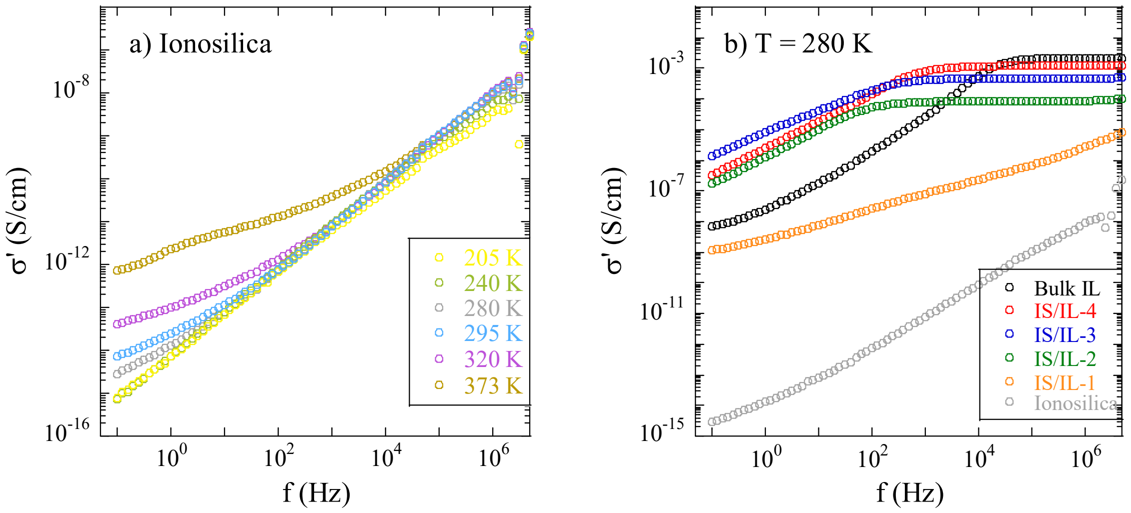

Figure S9. **(a)** Real part of the complex conductivity as a function of frequency at selected temperatures during heating for the ionosilica scaffold. **(b)** Comparison of samples with different confinement degrees at 280 K during heating.

The comparison between ε″ and the dc-free ε″ (calculated according to eq. 1 in the article) is shown in Figure S10 for each sample. The dc-free data are significantly lower than ε″ in the high-frequency range due to the suppression of the dc-conductivity contribution for all samples except IS/IL-1 (Figure S10-e). In this case, the two quantities overlap within the experimental uncertainty, indicating a negligible dc contribution. The main sources of uncertainty in BDS measurements arise from electrode/sample

contact quality, temperature stability, and uncertainties in sample geometry. In the present work, these effects were minimized through carefully controlled experimental conditions and do not affect the overall conclusions. Note that the superposition between ε″ and the dc-free ε″ is recovered for all samples in the low-frequency range, where electrode polarization becomes dominant.

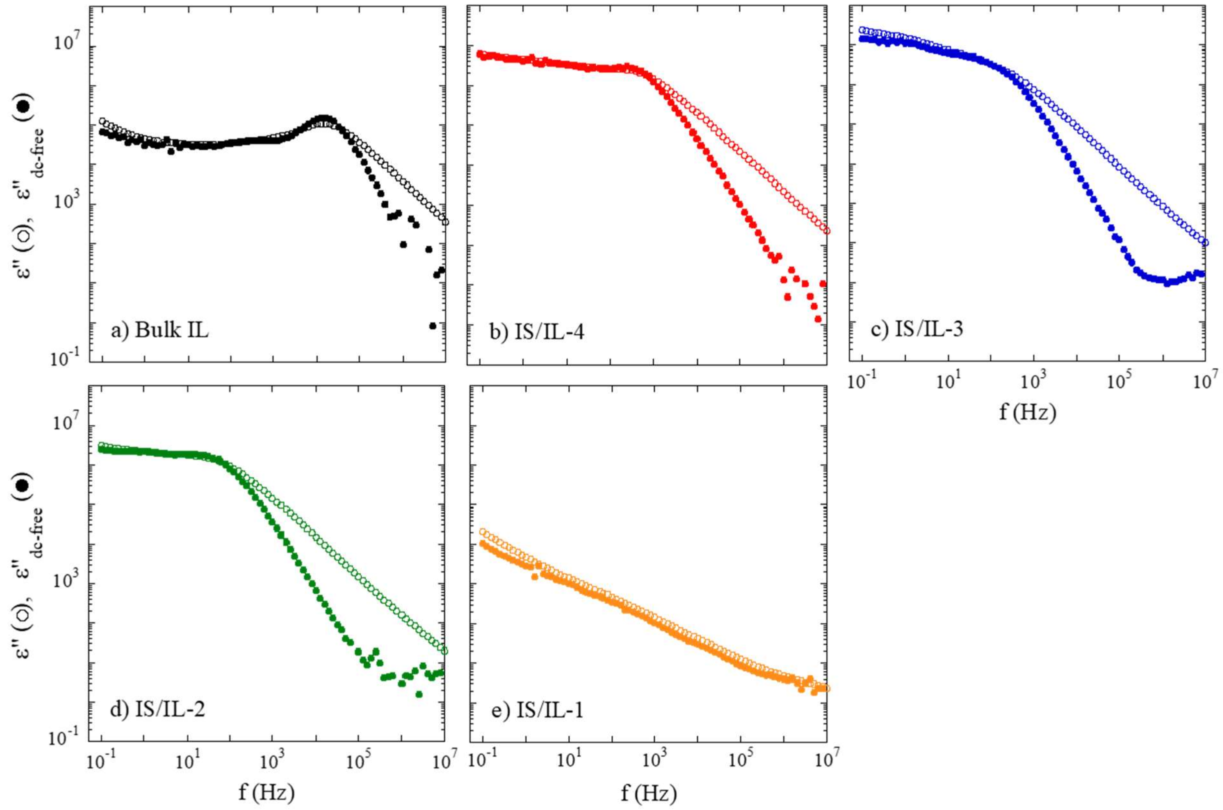

Figure S10. Frequency dependence of the imaginary part of the complex permittivity, ε'' (open symbols), and of the dc-free contribution, $\varepsilon''_{dc\text{-}free}$ (solid symbols), at 280 K for IS/IL samples with different degrees of confinement, as indicated in panels **(a)**, **(b)**, **(c)**, **(d)** and **(e)**.

Figure S11a reports the dependence of log(σ_{dc}) on the ionic liquid content in IS/IL composites at 205 K (Figure 4c) and 280 K (Figure S9b). A linear decrease is observed as the IL fraction is reduced, until IS/IL-2. Then, at the lowest IL loading (IS/IL-1), the dc conductivity deviates significantly from this linear trend, falling well below the expected value. Note that for IS/IL-1, where no dc contribution is detected, the conductivity value measured at the lowest frequency at each temperature is reported as an upper estimate of σ_{dc}. The same conductivity values are plotted in Figure S11b as a function of the mesopore fraction, which increases with decreasing IL content ($\Phi_{meso} = \Phi_{IL} - \Phi_{macro}$).

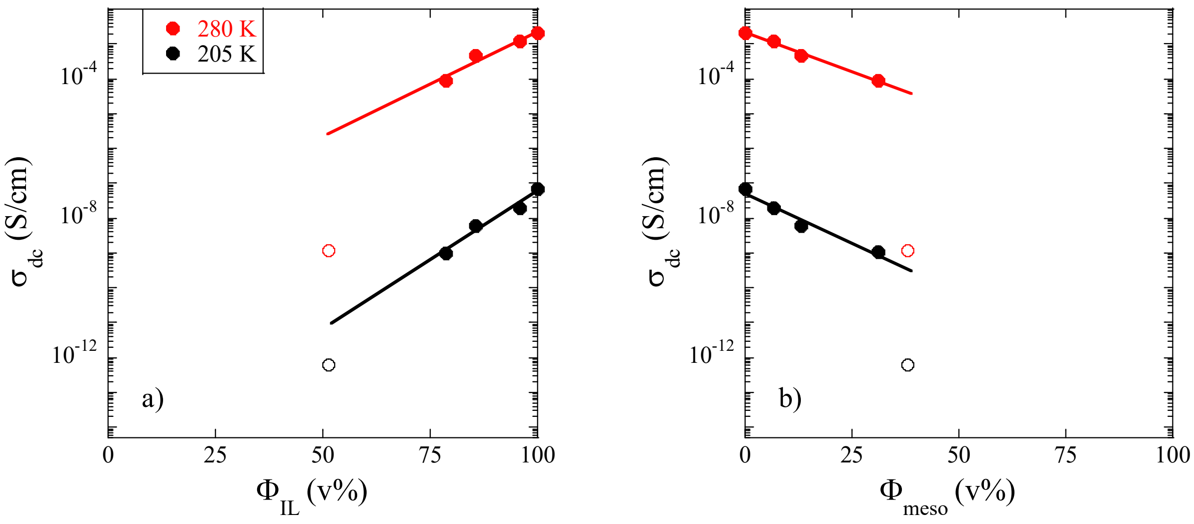

Figure S11. Evolution of the dc conductivity as a function of the IL volume fraction **(a)** and the mesopore volume fraction **(b)** at 205 K and 280 K, as indicated in the legend. The solid lines represent linear fits of log(σ_{dc}) to all data points except IS/IL-1, which is shown with open symbols (see text for details).

Figure S12 shows the temperature dependence of the ionic dc conductivity in IL and IS/IL composites. In many glass-forming liquids and ionic liquids, this dependence is non-Arrhenius, and it is commonly described by the empirical Vogel-Fulcher-Tammann (VFT) equation rather than by a simple Arrhenius law with a constant activation energy. The VFT law is written as

$$\sigma_{dc} = \sigma_0 \exp\left(\frac{-B}{T-T_0}\right) \qquad \text{S2}$$

where σ_0 is the pre-exponential factor, B is an activation parameter related to the energy barrier for ionic transport, and T_0 is the Vogel temperature, typically located below T_g. As can be seen in Figure S12, the experimental data for all samples are well described by the VFT law.

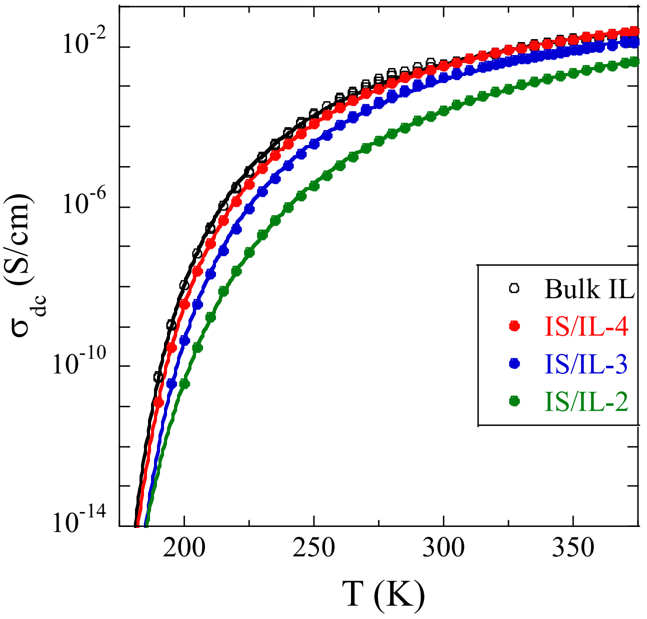

Figure S12. Temperature dependence of the dc conductivity for the bulk ionic liquid and under three different confinement conditions (composites IS/IL), as indicated in the legend, measured upon cooling. The solid lines correspond to fits of the VFT law (eq. S2).

The fitted parameters are summarized in Table S3. For the bulk IL, the B parameter (799 K) is in good agreement with literature values, which typically range from 537 to 934 K, [5-12] with an average value of 732 K. A comparison of the temperature dependence of pure BMIM-TFSI with selected literature data is shown in Figure S13.

Table S3. Parameters of the VFT law (eq. S2) for the dc conductivity obtained on cooling.

Sample	σ_0 (S/cm)	B (K)	T_0 (K)
Bulk IL	0.9 ± 0.3	798 ± 53	156.3 ± 2.1
IS/IL-4	1.8 ± 0.5	923 ± 50	154.0 ± 1.7
IS/IL-3	1.3 ± 0.4	995 ± 62	154.5 ± 2.3
IS/IL-2	2.2 ± 0.5	1440 ± 52	141.6 ± 1.7

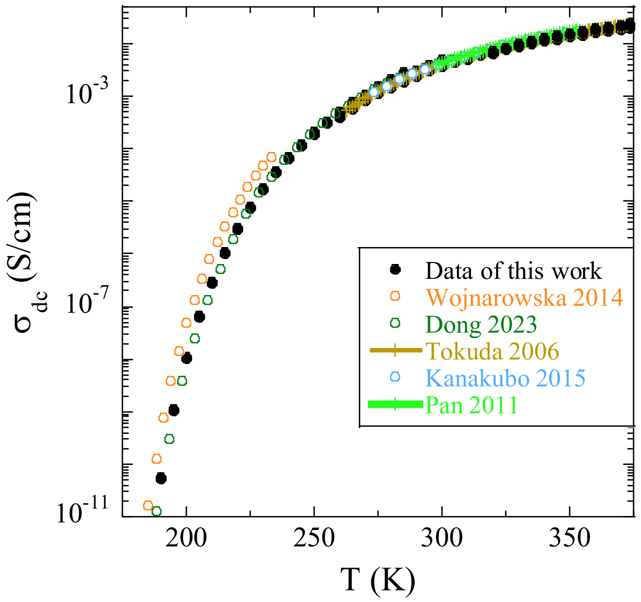

Figure S13. Temperature dependence of the dc conductivity of pure BMIM-TFSI measured during cooling (black circles) and compared with literature data [5-9] as indicated in the legend.

Figure S14 compares conductivity data measured at an intermediate temperature between the crystallization and melting of BMIM-TFSI for the different IS/IL composites. It highlights the impact of crystallization on the conductivity during heating, where the conductivity strongly decreases and the dc plateau is poorly defined.

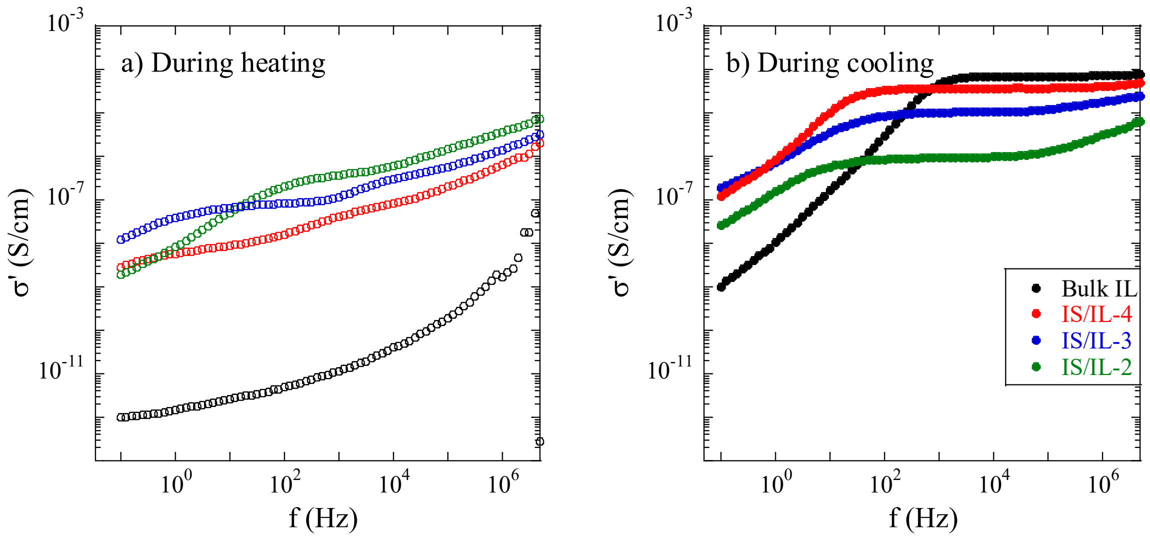

Figure S14. Real part of the complex conductivity as a function of frequency at 240 K, a temperature between the crystallization and melting temperatures of the ionic liquid, for samples with different confinement degrees during heating **(a)** and cooling **(b)**.

5. Additional BDS data – Structural relaxation

The VFT law for the structural relaxation time is written as

$$\tau_\alpha = \tau_0 \exp\left(\frac{B}{T - T_0}\right) \qquad S3$$

The VFT parameters allow the calculation of the fragility parameter, m, as defined by Böhmer et al. [13]

$$m = \frac{B\,T_g}{\ln(10)(T_g - T_0)^2} \qquad S4$$

m quantifies the steepness of the temperature dependence of the structural relaxation near T_g. The VFT and fragility parameters obtained are reported in Tables S4 and S5, respectively.

Table S4. Parameters of the VFT law (eq. S3) for the structural relaxation time, τ_α.

Sample	Log(τ_0 [s])	B (K)	T_0 (K)
Bulk IL	-12.1 ± 0.3	662 ± 55	161.0 ± 1.4
IS/IL-4	-12.1 ± 0.3	821 ± 110	156.7 ± 1.6
IS/IL-3	-11.9 ± 0.3	877 ± 122	155.0 ± 1.6
IS/IL-2	-11.9 ± 0.3	966 ± 102	153.8 ± 1.1
IS/IL-1	-12.1 ± 0.3	1186 ± 132	153.9 ± 1.7

Table S5. Fragility parameter (eq. S4) and T_g^{BDS} corresponding to the temperature at which τ_α = 100 s in eq. S3. T_g^{DSC} values correspond to the calorimetric T_g obtained by DSC during heating (3 K/min) and cooling (20 K/min).

Sample	T_g^{BDS} (K)	m	T_g^{DSC} (K) on heating	T_g^{DSC} (K) on cooling
Bulk IL	181.4 ± 0.2	125 ± 20	187.6 ± 0.1	187.3 ± 0.1
IS/IL-4	182.0 ± 0.3	101 ± 19	188.5 ± 0.1	188.5 ± 0.1
IS/IL-3	182.4 ± 0.2	93 ± 17	188.0 ± 0.1	187.6 ± 0.1
IS/IL-2	183.9 ± 0.1	85 ± 11	189.2 ± 0.1	190.1 ± 0.1
IS/IL-1	190.4 ± 0.2	74 ± 11	191.6 ± 0.1	193.2 ± 0.1

For comparison with the BDS T_g determined at τ_α = 100 s, the T_g's obtained by TMDSC at a heating rate of 3 K/min are reported in Table S5. In addition, T_g values determined during cooling at a rate of 20 K/min have been included. As the cooling segments were not performed in modulated DSC mode, T_g was defined in this case (cooling) as the inflection point of the total heat flow signal, rather than from the reversible heat capacity as used for the heating scans.

References

[1] P. Sippel, S. Krohns, D. Reuter, P. Lunkenheimer, A. Loidl, Importance of reorientational dynamics for the charge transport in ionic liquids, Physical Review E 98(5) (2018) 052605.

[2] C. Krause, J.R. Sangoro, C. Iacob, F. Kremer, Charge Transport and Dipolar Relaxations in Imidazolium-Based Ionic Liquids, The Journal of Physical Chemistry B 114(1) (2010) 382-386.

[3] A. Rivera, E.A. Rössler, Evidence of secondary relaxations in the dielectric spectra of ionic liquids, Physical Review B 73(21) (2006) 212201.

[4] S. Sharma, J. Oberdisse, J.G. Alauzun, P. Dieudonné-George, T. Bizien, C. Akkaya, P. Hesemann, A.-C. Genix, Controlled formation of multi-scale porosity in ionosilica templated by ionic liquid, Nanoscale 16(12) (2024) 6053-6067.

[5] Y. Dong, M. Steinhart, H.-J. Butt, G. Floudas, Conductivity of Ionic Liquids In the Bulk and during Infiltration in Nanopores, The Journal of Physical Chemistry B 127(31) (2023) 6958-6968.

[6] Y. Pan, L.E. Boyd, J.F. Kruplak, W.E. Cleland, J.S. Wilkes, C.L. Hussey, Physical and Transport Properties of Bis(trifluoromethylsulfonyl)imide-Based Room-Temperature Ionic Liquids: Application to the Diffusion of Tris(2, 2′-bipyridyl)ruthenium(II), Journal of The Electrochemical Society 158(1) (2011) F1.

[7] M. Kanakubo, K.R. Harris, N. Tsuchihashi, K. Ibuki, M. Ueno, Temperature and Pressure Dependence of the Electrical Conductivity of 1-Butyl-3-methylimidazolium Bis(trifluoromethanesulfonyl)amide, Journal of Chemical & Engineering Data 60(5) (2015) 1495-1503.

[8] H. Tokuda, K. Ishii, M.A.B.H. Susan, S. Tsuzuki, K. Hayamizu, M. Watanabe, Physicochemical Properties and Structures of Room-Temperature Ionic Liquids. 3. Variation of Cationic Structures, The Journal of Physical Chemistry B 110(6) (2006) 2833-2839.

[9] Z. Wojnarowska, G. Jarosz, A. Grzybowski, J. Pionteck, J. Jacquemin, M. Paluch, On the scaling behavior of electric conductivity in [C4mim][NTf2], Physical Chemistry Chemical Physics 16(38) (2014) 20444-20450.

[10] Y. Fu, X. Cui, Y. Zhang, T. Feng, J. He, X. Zhang, X. Bai, Q. Cheng, Measurement and Correlation of the Electrical Conductivity of the Ionic Liquid [BMIM][TFSI] in Binary Organic Solvents, Journal of Chemical & Engineering Data 63(5) (2018) 1180-1189.

[11] E.A. Arkhipova, A.S. Ivanov, K.I. Maslakov, S.V. Savilov, V.V. Lunin, Effect of cation structure of tetraalkylammonium- and imidazolium-based ionic liquids on their conductivity, Electrochimica Acta 297 (2019) 842-849.

[12] Y. Dong, H. He, K. Kapil, M. Steinhart, K. Matyjaszewski, H.-J. Butt, G. Floudas, Tethered Cation Size Affects the Imbibition of Polymerized Ionic Liquids and the Ionic Conductivity in Nanopores, Macromolecules 58(14) (2025) 7534-7543.

[13] R. Böhmer, K.L. Ngai, C.A. Angell, D.J. Plazek, Nonexponential relaxations in strong and fragile glass formers, The Journal of chemical physics 99(5) (1993) 4201-4209.
